# Population-level geometric filtering generates motion-coherence selectivity in the retina

**DOI:** 10.1126/sciadv.aeg7846

**Published:** 2026-08-19

**Authors:** Pratyush Ramakrishna, Andrew Jo, Liam McCoy, Josh L. Morgan, Daniel Kerschensteiner

**Affiliations:** ^1^ John F. Hardesty, MD Department of Ophthalmology and Visual Sciences, Washington University in St. Louis, St. Louis, MO, USA.; ^2^ Department of Biomedical Engineering, Washington University in St. Louis, St. Louis, MO, USA.; ^3^ Department of Neuroscience, Washington University in St. Louis, St. Louis, MO, USA.; ^4^ Bright Center for Human Vision, Washington University in St. Louis, St. Louis, MO, USA.

## Abstract

Object-motion–sensitive (OMS) circuits must ignore self-motion while remaining sensitive to independently moving objects. We show that the mouse retina solves this by geometrically filtering motion coherence. Correlative light and electron microscopy reveal that TH2 amacrine cells (GABAergic Type-2 wide-field amacrine cells in the TH-Cre mouse strain) combine a dense proximal arbor with sparse radiating distal neurites, distributing synapses uniformly across both compartments. Two-photon imaging and compartmental modeling establish that sparse inhibition and voltage-gated sodium channels allow this bipartite arbor to multiplex signals: Proximal dendrites compute locally, whereas distal neurites integrate over hundreds of micrometers. A population model built from this architecture predicts a daisy-shaped inhibitory surround for OMS ganglion cells (W3/Ultra-High-Definition), with a circular near-surround and radially orientation-selective far-surround subunits. Patch-clamp recordings confirmed this organization and revealed that the surround suppresses coherent global motion while preserving object-motion detection, even amid high densities of independently moving objects. Thus, single-cell morphology and dendritic processing scale through plexus geometry to implement motion-coherence selectivity.

## INTRODUCTION

A fundamental axiom of neuroscience is that dendritic morphology constrains neuronal computation (*1*). Since the time of Cajal (*2*), the immense diversity of dendritic arbors has been assumed to support distinct functional roles (*3*, *4*). However, demonstrating how specific dendritic architectures implement specific algorithmic operations remains a substantial challenge. While simple linear integration is well described by passive cable theory (*5*), understanding how neurons exploit complex branching patterns, active conductances, and synaptic distributions to perform nonlinear computations requires bridging levels of organization that are rarely studied together. For single neurons, this means bridging ultrastructural connectivity, dendritic biophysics, and dendritic computation (*6*–*9*). Even more challenging is the transition to neuronal populations, where the overlapping arbors of many neurons form a dense neuropil or plexus whose collective properties determine the computational landscape available to downstream targets and remain almost entirely unexplored.

Here, we provide an integrated account of how dendritic form generates circuit function, scaling from electron microscopy of single synapses to the geometry and computational logic of a neuronal population. We use the mammalian retina as a tractable model system to address this multiscale challenge, focusing on the construction of sensory surrounds (*10*–*13*). In the visual system, surrounds provide the context necessary for efficient coding and feature extraction. While simple surrounds linearly subtract mean intensity for efficient coding (*14*–*16*), complex surrounds extract higher-order features to support specific behavioral goals (*9*, *17*, *18*).

To reliably detect object motion (e.g., a predator or prey) amidst the global image motion caused by eye, head, or body movements, object-motion–sensitive (OMS) circuits rely on a complex surround (*17*, *19*–*22*) . OMS is often described as the ability to distinguish a small moving object from large-field image motion. However, this size-based definition is incomplete. Natural scenes can contain many independently moving objects, such that object motion can occupy a large fraction of the retinal image. A circuit that simply suppresses large or dense motion would therefore risk silencing object responses in clutter. The more general computation is to distinguish the coherence of motion: Coherent motion across the visual field, as occurs during self-motion, should be suppressed, whereas incoherent motion at similar density, arising from independently moving objects, should preserve object responses. Whether such motion-coherence selectivity exists in the retina and how OMS surround geometry could support it remain unknown.

Wide-field amacrine cells are primary architects of retinal surrounds (*4*, *13*, *22*, *23*). Their neurites span millimeters of the retinal surface, forming a dense, overlapping plexus that allows a single ganglion cell (retinal output neuron) to sample visual space far beyond its dendritic field. However, it remains unknown how the morphology of individual wide-field interneurons shapes the computational properties of this collective neuropil. Do these cells pool all inputs to generate a uniform output signal, or do their dendrites compartmentalize signals to create structured, nonlinear inhibitory receptive fields?

To answer this, we focused on TH2 amacrine cells (GABAergic Type-2 wide-field amacrine cells in the TH-Cre mouse strain), a conserved wide-field interneuron implicated in the cancelation of self-motion signals in OMS circuits (*21*–*26*). Using genetically targeted correlative light and electron microscopy (CLEM), we first reveal that TH2 cells have a notable bipartite morphology, segregating the arbor into a symmetric, densely branched proximal compartment and a sparse, radially oriented distal compartment.

By combining two-photon calcium imaging with compartmental modeling, we demonstrate that this morphological division, supported by voltage-gated sodium conductances and sparse inhibition, enables signal multiplexing: Proximal dendrites compute locally, whereas distal neurites act as long-range transmission lines. We then incorporate these biophysical constraints into a population model of the TH2 plexus. The model predicts that the inhibitory surround of downstream OMS ganglion cells [W3/Ultra-High-Definition (UHD)] is not spatially uniform but instead decomposes into a circular near-surround and radially orientation-selective far-surround subunits. Two-photon imaging and patch-clamp recordings confirmed these predictions. This daisy-shaped geometry further predicted motion-coherence selectivity. Coherent global motion aligns with the preferred radial axes of a subset of far-surround subunits—those parallel to the motion trajectory—and recruits them synchronously, producing strong summed inhibition of W3/UHD output. In contrast, under incoherent motion at matched visual-field occupancy, most objects move along nonradial trajectories. The few objects that do engage a far-surround subunit’s preferred axis do so at uncorrelated times, yielding weak, asynchronous inhibition that spares responses to independently moving objects. Targeted recordings confirmed this prediction. We refer to this transformation of motion coherence into differential inhibition by single-cell and population-level circuit geometry as geometric filtering.

## RESULTS

### A bipartite dendritic architecture in TH2 amacrine cells

**Fig. 1.**
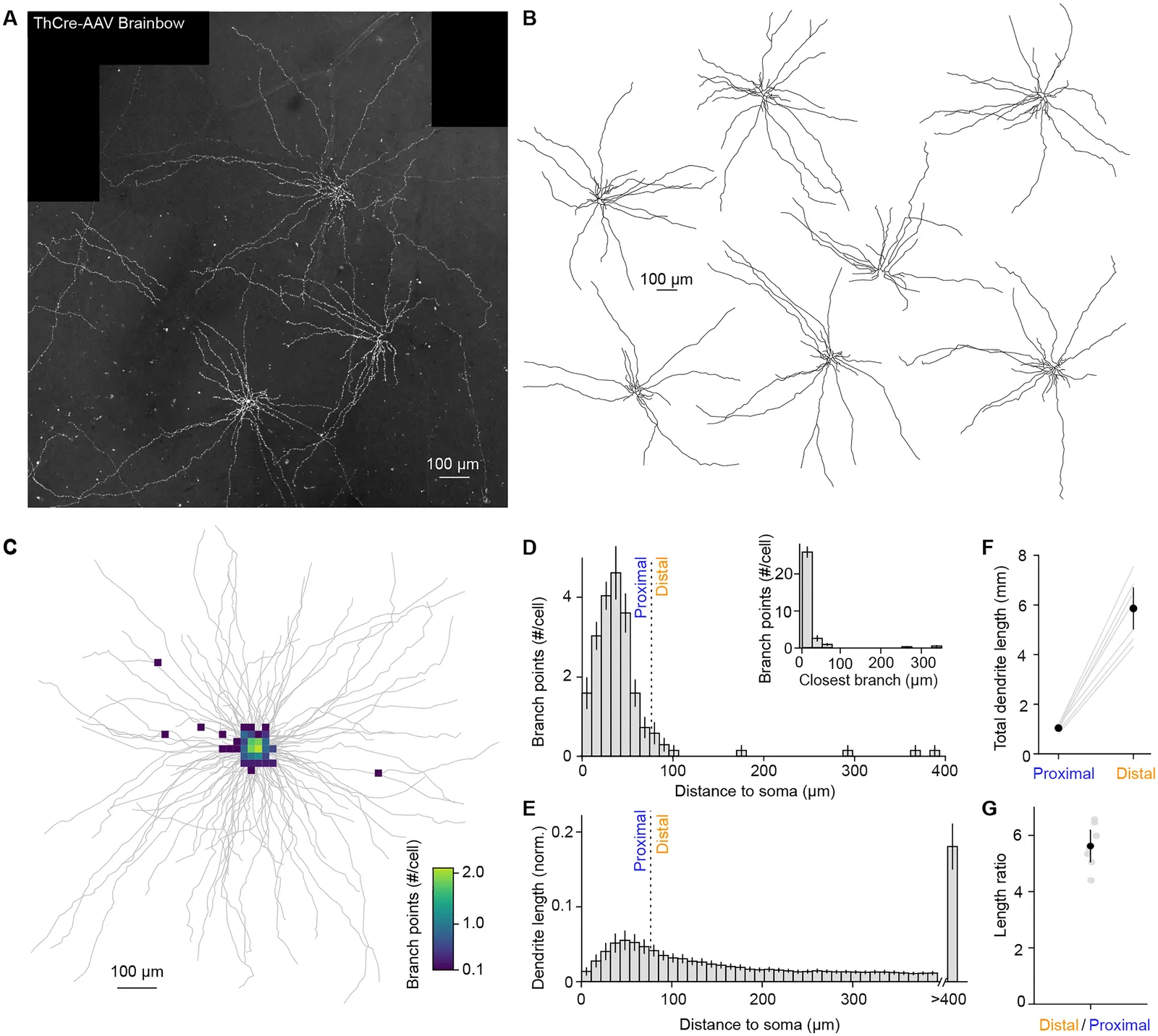
Bipartite dendritic architecture in TH2 amacrine cells. (**A**) Sparse AAV-Brainbow labeling of TH2 amacrine cells in TH-Cre mice reveals wide-field arbors in the inner plexiform layer (IPL). The representative image shows a mixture of yellow and teal fluorescent protein signals. (**B**) Manual skeletonization of complete TH2 dendritic arbors. Throughout this figure, *n* = 7 cells from four mice. (**C**) Superimposed TH2 dendritic skeletons with a two-dimensional (2D) histogram of branch point positions, showing dense proximal branching near the soma and long, unbranched dendrites extending distally. (**D**) Distribution of path lengths from the soma to dendritic branch points across reconstructed cells. Ninety-five percent of branch points lie within 76.1 μm of the soma; we therefore define segments within this radius as proximal and those beyond as distal. Inset: Distribution of interbranch distances across the arbor, showing tightly clustered branch points (95% ≤ 44.1 μm). Error bars indicate mean ± SEM across cells. (**E**) Cumulative dendritic length as a function of distance from the soma across seven cells, illustrating the relative contributions of proximal and distal compartments. Error bars indicate mean ± SEM across cells. norm., normalized per cell. (**F**) Total proximal and distal dendritic length per cell. (**G**) Ratio of distal and proximal dendritic length defines the probability that synaptic partners encounter distal versus proximal dendrites in the TH2 plexus. Partners are 5.62-fold [95% confidence interval (CI): 4.90, 6.34] more likely to encounter distal dendrites. Together, these data reveal a bipartite architecture consisting of a compact, highly branched proximal region and an expansive distal region formed by long, radially oriented, sparsely branched dendrites.

To determine how dendritic form influences wide-field processing, we first characterized the morphology of TH2 amacrine cells. We sparsely labeled these interneurons in TH-Cre mice (*27*) using Brainbow adeno-associated viruses (AAVs) (*28*) and reconstructed seven complete arbors from confocal images (Fig. 1, A to C). The skeletons revealed a notable bipartite organization: a compact, highly branched proximal zone surrounding the soma; and a vast distal zone composed of long (>500 μm), unbranched dendrites radiating outward (*29*). Quantitative analysis of the branching pattern defined a sharp boundary between these compartments. Ninety-five percent of branch points were located within 76.1 μm of the soma. Processes extending past this boundary were largely unbranched (Fig. 1D). Within the proximal zone, branch points were tightly clustered, with 95% of interbranch distances ≤ 44.1 μm (Fig. 1D, inset). In later experiments, this sharp difference in branch density allowed us to classify dendrites as proximal and distal in the absence of information about the soma position.

On average, TH2 arbors contained 1.04 mm [95% confidence interval (CI): 0.94, 1.13 mm] of proximal dendrite versus 5.86 mm [5.03, 6.69 mm] of distal dendrite (Fig. 1, E and F). Consequently, a synaptic partner encountering a TH2 neurite in the plexus is 5.62-fold [5.08, 6.14] more likely to contact a distal dendrite than a proximal one (Fig. 1G). These data establish that TH2 arbors are organized into two structurally distinct compartments: a compact, highly branched proximal region and an expansive distal region formed by long, radially oriented, sparsely branched dendrites. Proximal and distal compartments are not separate dendrites but successive segments of the same dendrites; long unbranched distal dendrites emerge from the terminal branches of a densely branched proximal core rather than from the soma directly, with most terminal tips separated from the soma by three to five successive branch points (fig. S1).

### Uniform input and output synapse distributions across the bipartite TH2 arbors

**Fig. 2.**
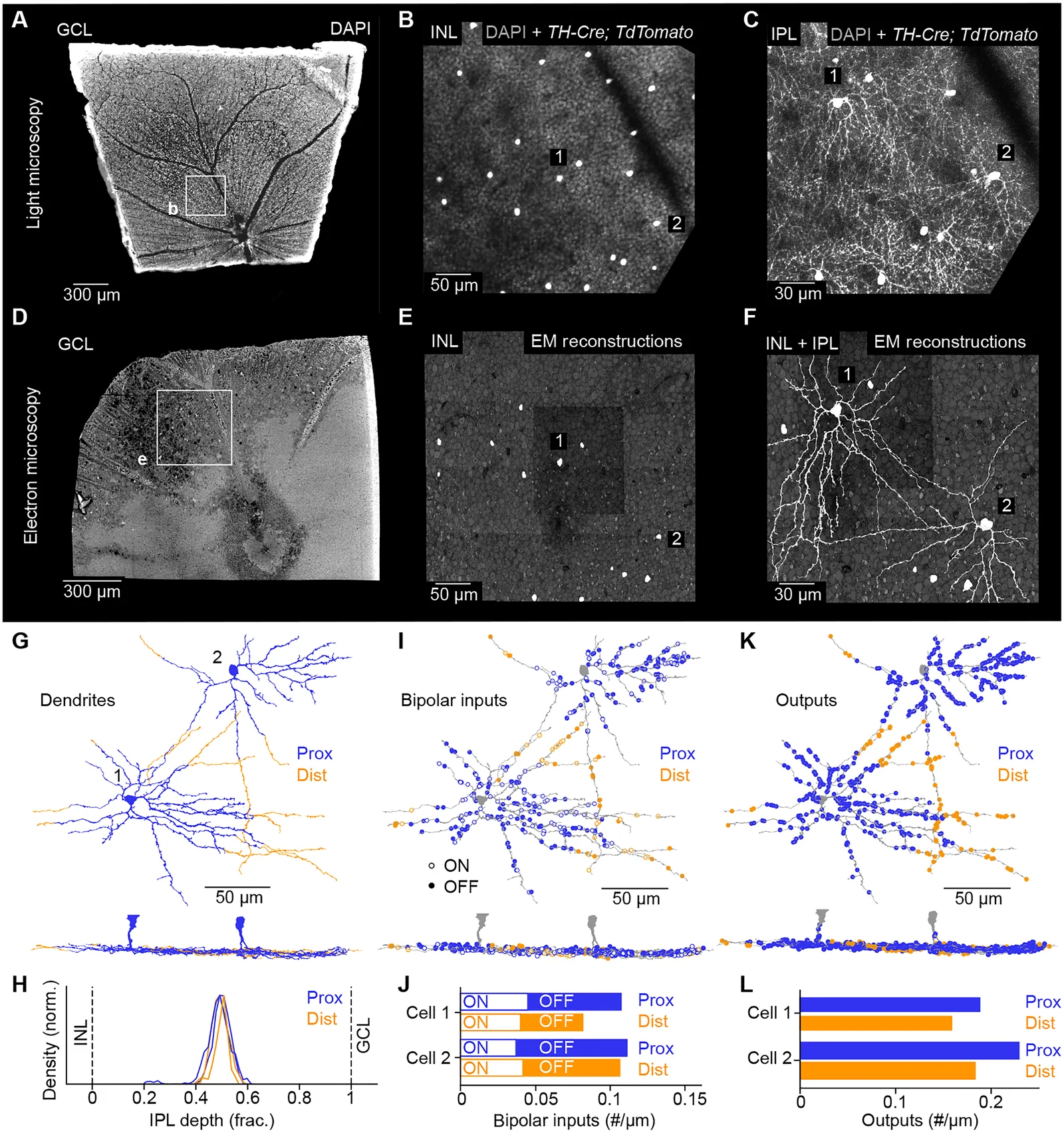
Uniform input and output synapse distributions and selective bipolar input to TH2 amacrine cells. (**A** to **C**) Confocal stack of a TH2-Cre; Ai9 retina showing tdTomato-labeled TH2 cells and 4′,6-diamidino-2-phenylindole (DAPI)–stained nuclei in the ganglion cell layer (GCL; A), inner nuclear layer (INL; B), and IPL (C), respectively. (**D** to **F**) A serial-section 3D electron microscopy (3DEM) volume registered to the confocal dataset allows for the identification and reconstruction of multiple TH2 amacrine cells. (**G** and **H**) Ultrastructural reconstructions of two TH2 cells within the 300 × 300 × 48 μm 3DEM volume (*n* = 2 cells from one mouse). Dendrites stratify narrowly in the center of the IPL and are classified as proximal or distal based on the interbranch distance criterion established in Fig. 1. frac., fractional; Prox, proximal; Dist, distal. (**I**) Bipolar cell input synapse (i.e., ribbon synapse) densities along proximal and distal dendrites for the two reconstructed cells. Bipolar cell inputs (excitatory) are uniformly distributed across compartments (proximal: cell 1 = 0.11 and cell 2 = 0.11 syn/μm; distal: 0.08 and 0.10 syn/μm). syn, synapses. (**J**) Both compartments receive mixed ON and OFF bipolar input, with a bias toward OFF bipolar input (ON/OFF ratio proximal: 0.73 and 0.5; distal: 0.97 and 0.65). (**K** and **L**) Output synapse densities are similarly uniform across proximal and distal dendrites (proximal: 0.20 and 0.23 syn/μm; distal: 0.17 and 0.20 syn/μm).

The bipartite morphology of TH2 cells resembles that of polyaxonal amacrine cells, which are thought to segregate inputs and outputs into distinct dendritic and axonal compartments (*4*, *30*–*32*). To test whether TH2 cells exhibit such segregation, we performed CLEM (*33*). We identified individual TH2 cells in a TH-Cre; Ai9 retina using confocal microscopy and subsequently reconstructed the same neurons in a serial-section three-dimensional electron microscopy (3DEM) volume (Fig. 2, A to F, a; 300 × 300 × 48 μm). We reconstructed two TH2 cells and annotated all synapses along their dendrites within the volume.

Both cells stratified narrowly in the center of the inner plexiform layer (IPL; Fig. 2, G and H). Using the interbranch distance criterion defined above, we classified reconstructed dendrites as proximal or distal. Although each TH2 arbor extended beyond the 3DEM field of view, the volume captured substantial portions of both compartments, allowing comparison of synapse densities. Contrary to the hypothesis of segregated processing, we found a uniform distribution of synaptic inputs and outputs across the arbor (Fig. 2, I to L) (*29*). Excitatory ribbon synapses from bipolar cells innervated proximal and distal compartments at similar densities (proximal: cell 1 = 0.11 and cell 2 = 0.11 syn/μm; distal: 0.08 and 0.10 syn/μm; Fig. 2, I and J) (*34*). OFF bipolar cell inputs were more numerous than ON bipolar inputs (ON/OFF ratio proximal: 0.73 and 0.5; distal: 0.97 and 0.65). Similarly, output synapses were distributed uniformly across both compartments (proximal: 0.20 and 0.23 syn/μm; distal: 0.17 and 0.20 syn/μm; Fig. 2, K and L). This uniform distribution of excitatory inputs and inhibitory outputs across compartments was preserved when we applied an alternative morphological definition to segment the bipartite arbor (fig. S2). Although the 3DEM volume samples only a portion of the full TH2 arbor, prior light-microscopy analyses of synaptic markers across complete TH2 arbors are consistent with a homogeneous distribution at the population level (*29*), supporting the extrapolation of our compartment-specific densities to the full arbor. Thus, despite their specialized morphology, TH2 proximal and distal compartments function as integrated input-output units rather than segregated dendritic and axonal domains.

### Distal and proximal TH2 dendrites differ in their receptive fields

**Fig. 3.**
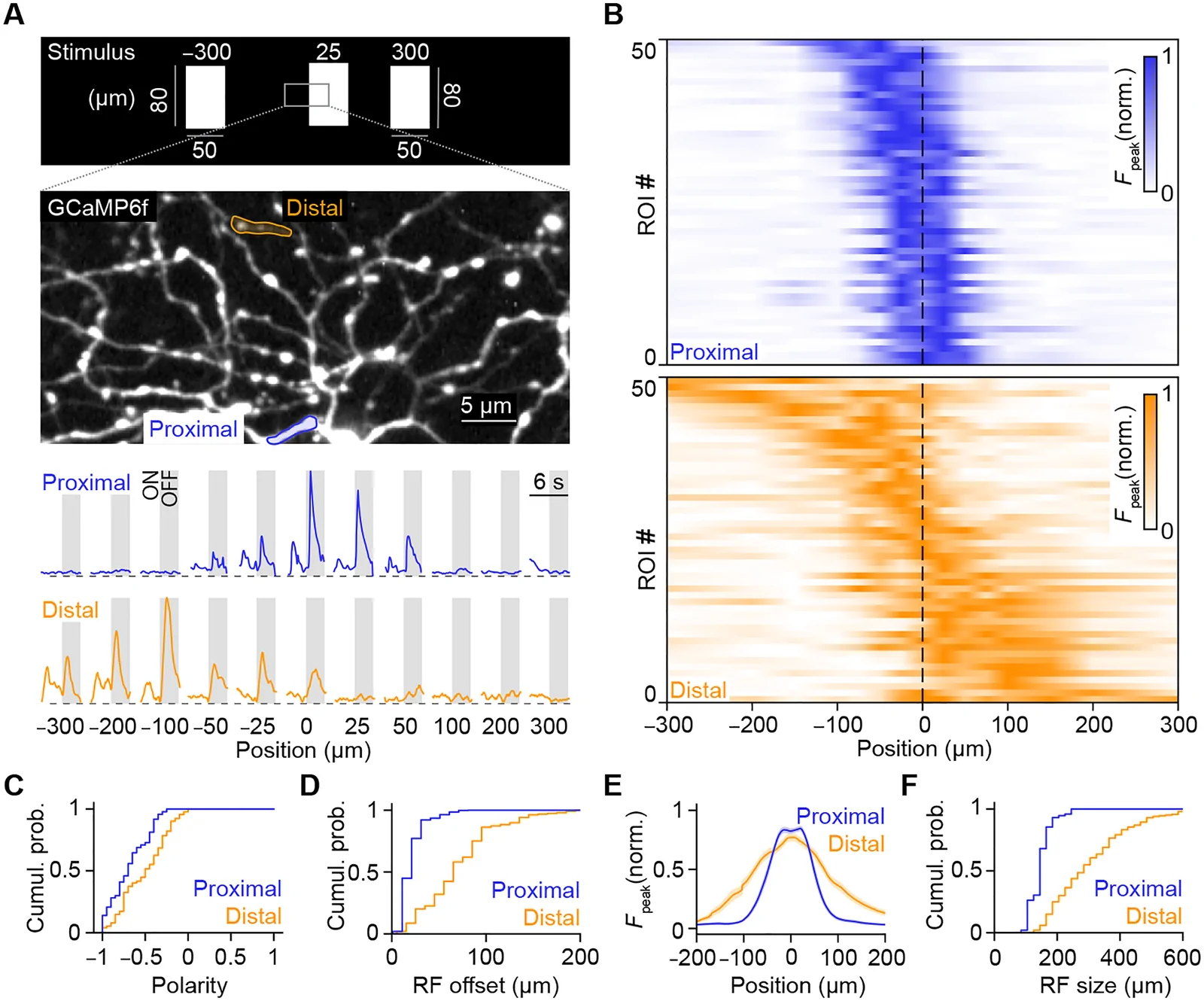
Distal and proximal TH2 dendrites differ in their receptive fields. (**A**) Two-photon calcium imaging of GCaMP6f-expressing TH2 dendrites in TH-Cre; Ai148 retinas. Proximal (blue) and distal (orange) regions of interest (ROIs) were segmented using the interbranch distance threshold from Fig. 1 (fig. S3). Receptive fields (RF) were mapped with a vertical bar (80 × 50 μm) flashed from −300 to +300 μm along the horizontal axis. Traces show Δ*F*/*F* responses to ON and OFF phases. Proximal ROIs respond to bars near the imaging center, whereas horizontally aligned distal ROIs also respond to distant bars. (**B**) Peak-normalized OFF responses across bar positions for the 50 most reliable proximal and horizontally aligned distal ROIs. Because the stimulus varied only along the horizontal axis, only horizontally oriented distal dendrites were included in the primary analysis; ON responses and vertically oriented distal dendrites are shown in fig. S3. Throughout, *n* = 11 imaging regions from four mice. (**C**) Cumulative probability (Cumul. prob.) distributions of polarity indices, indicating OFF-dominated responses in both compartments (proximal: −0.71 [−0.77, −0.64]; distal: −0.53 [−0.60, −0.45]). (**D**) Receptive field offsets (distance between each ROI’s response center of mass and its dendritic position). Proximal offsets are small (15.8 μm [11.6, 19.9]), whereas distal offsets are large (65.4 μm [53.1, 77.7]). (**E**) Mean OFF receptive field profiles aligned to their centers. (**F**) Receptive field sizes (full width at 25% of peak), showing compact proximal fields (137.0 μm [127.0, 147.0]) and ∼2-fold larger distal fields (ratio: 2.15 [1.75, 2.60]; 294.1 μm [258.7, 329.5]). Proximal dendrites have small, locally centered receptive fields, whereas distal dendrites have large, displaced fields expected to deliver wide-field inhibition to downstream targets.

Given the uniform synaptic density, we asked whether the bipartite branching pattern alone is sufficient to generate compartment-specific visual responses. We expressed GCaMP6f in TH2 cells (TH-Cre; Ai148 mice) and used two-photon calcium imaging to map receptive fields across the arbor. We presented flashing bars at varying horizontal positions from the recording site and segmented regions of interest (ROIs) in proximal and distal dendrites (Fig. 3A and fig. S3). Because the mapping stimulus varied along a single (horizontal) axis, we restricted our main analysis to distal ROIs whose local dendritic orientation falls within ±45° of horizontal; vertically aligned distal dendrites sample the stimulus along their short dimension and are shown separately in fig. S3.

This functional mapping revealed a dichotomy that paralleled the morphological division. Proximal dendrites exhibited spatially compact receptive fields centered near the recording site (Fig. 3B). In contrast, distal dendrites exhibited broad receptive fields that were sensitive to stimuli hundreds of micrometers away. Thus, while both compartments responded to ON and OFF stimuli (with a shared OFF bias; Fig. 3C), their spatial tuning differed significantly. We quantified the spatial offset between the dendritic ROI and its receptive field center (Fig. 3D and fig. S3): Proximal receptive fields were closely colocalized with their dendrites (offset: 15.4 μm [11.8, 19.8 μm]), whereas distal receptive fields were substantially displaced (offset: 64.9 μm [53.6, 77.3 μm]). Furthermore, distal receptive fields were about twice as wide as proximal receptive fields (294.1 μm [261.0, 329.0 μm] versus 137.0 μm [127.0, 147.0 μm]; Fig. 3, E and F). These results demonstrate that the TH2 arbor multiplexes visual signals: Proximal dendrites process local features, while distal dendrites integrate information from remote regions of the visual scene.

### Compartmental TH2 arbor model reproduces receptive field properties

**Fig. 4.**
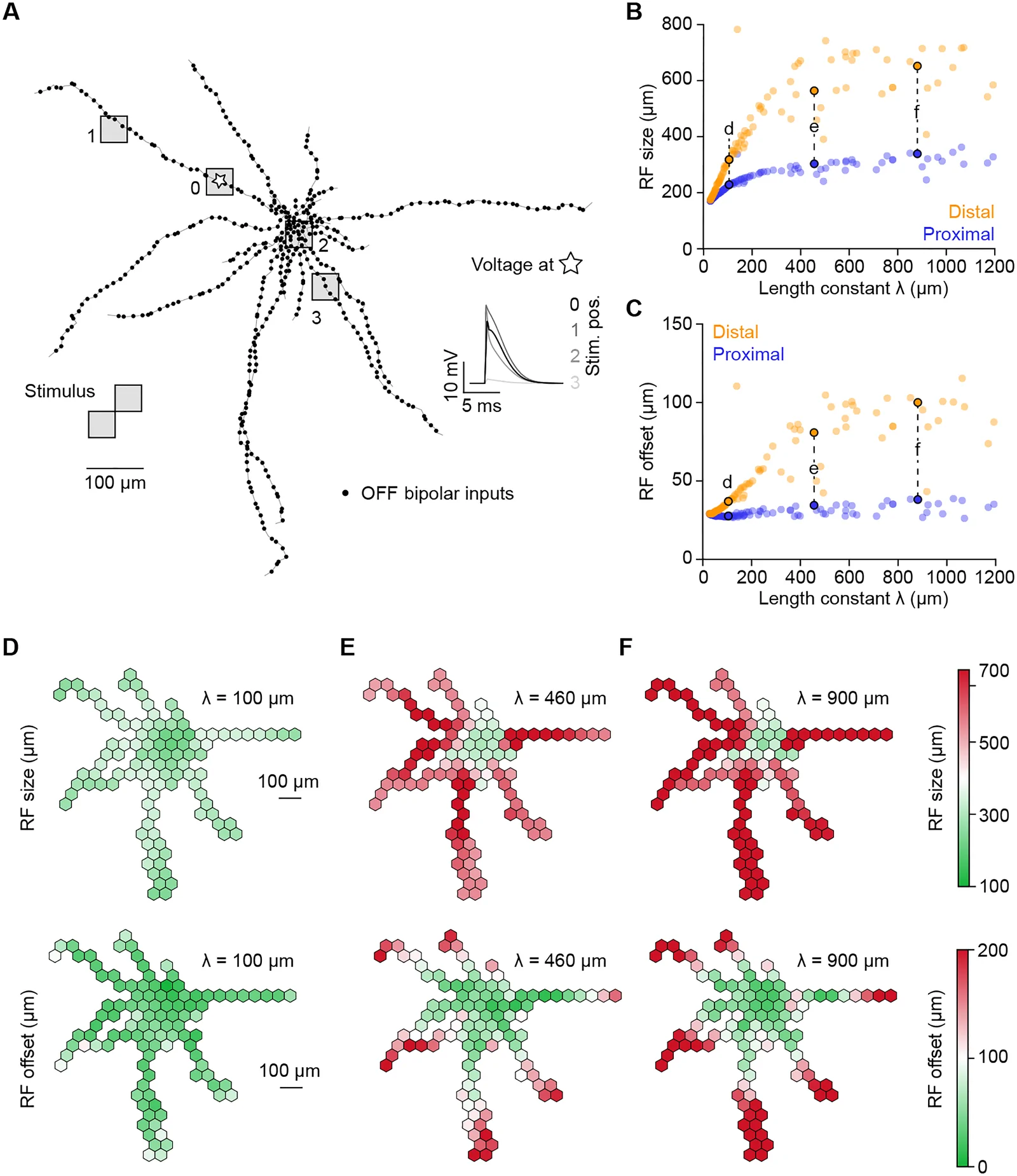
Compartmental TH2 arbor model reproduces proximal and distal receptive field properties. (**A**) Compartmental model of a TH2 amacrine cell in NEURON, with morphology from a representative confocal reconstruction and dendritic diameters from 3DEM. We focus on the functionally dominant OFF bipolar inputs, distributed at the measured density (0.064 syn/μm). Receptive fields are mapped by activating all OFF synapses within 50 × 50 μm squares tiling the arbor and measuring peak depolarization at dendritic recording sites. At the site marked by the star, activation at stimulus positions (Stim. pos.) 0 to 2 evokes robust depolarizations (inside the receptive field), whereas position 3 produces negligible depolarization (outside). (**B**) Receptive field sizes of proximal and distal dendrites (full width at 25% of peak) as a function of the electrotonic length constant λ. For each λ, synaptic weights are scaled to evoke ∼30-mV maximal depolarizations (*22*); parameter tuning is detailed in fig. S4. (**C**) Receptive field offsets (distance between each spatial voltage profile’s center of mass and the recording position) as a function of λ. (**D** to **F**) Receptive field properties across the arbor of an example cell. At λ = 100 μm (D), both compartments show compact, locally centered fields. At λ = 460 μm (E) and 900 μm (F), proximal dendrites retain compact fields, while distal dendrites develop large, displaced fields. Long-distance integration of uniformly distributed inputs across the bipartite arbor is therefore sufficient to establish the distinct receptive field properties of the two compartments over a broad range of length constants (λ = 300 to 1200 μm).

To define the precise biophysical rules that translate this bipartite morphology into dual-channel signal processing, we built an anatomically constrained model of a TH2 cell in NEURON (*35*). A confocally reconstructed skeleton provided the branching pattern; dendritic diameters were assigned from our 3DEM measurements (Fig. 4A). Focusing on OFF bipolar cell input, we distributed synapses across dendrites at the density measured in the 3DEM dataset (0.064 syn/μm). We then simulated receptive field mapping by activating synapses in tiling patches and recording voltage responses across the arbor.

We varied the electrotonic length constant (λ) by changing passive membrane conductance (fig. S4). At short length constants (e.g., λ = 115 μm), signals dissipated rapidly, resulting in small, local receptive fields in both compartments (Fig. 4, B to D). However, as λ increased (λ = 300 to 1200 μm), the unbranched distal dendrites acted as efficient transmission lines in the model, allowing signals to propagate over long distances and generating large, displaced receptive fields. In contrast, the dense branching of the proximal arbor continued to promote charge dissipation, keeping proximal receptive fields compact (Fig. 4, B, C, E, and F). Thus, our modeling indicates that the bipartite branching pattern, when supported by adequate electrotonic propagation, naturally generates the observed dual-channel processing architecture. A model with an inverted branching pattern (sparse proximal and dense distal) failed to replicate this physiology, instead generating local receptive fields in the dense distal tips (fig. S5).

### Sparse inhibition enables long-distance voltage propagation in TH2 dendrites

**Fig. 5.**
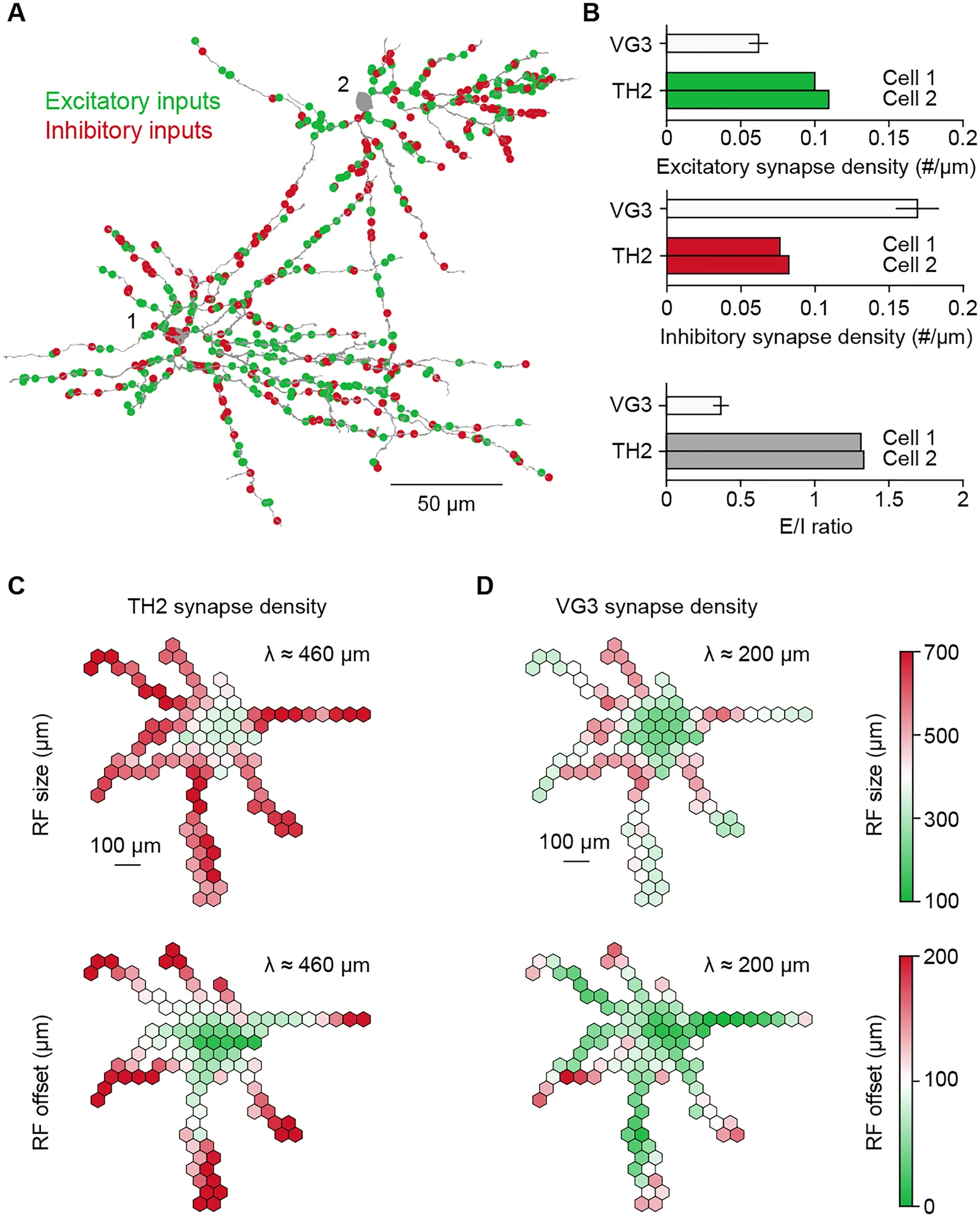
Sparse inhibition enables long-distance voltage propagation in TH2 dendrites. (**A**) 3DEM reconstructions of two TH2 amacrine cell dendrites with excitatory (ribbon) and inhibitory (conventional) synapses annotated. (**B**) Quantification of excitatory and inhibitory input shows an excitation-biased configuration in TH2. For comparison, we previously reported an inhibition-biased configuration in VGluT3 (VG3) amacrine cells (*39*). Excitation/inhibition (E/I) ratios were 1.3 and 1.31 for the two reconstructed TH2s and 0.37 ± 0.04 for VG3 (*n* = 6 cells from one mouse). (**C**) Compartmental TH2 model with inhibitory synapses added at measured densities. Membrane conductances were tuned to yield λ ≈ 460 μm and reproduce the proximal-distal receptive field dichotomy. (**D**) Replacing TH2 synapse densities with VG3 synapse densities in the same TH2 morphology collapses distal receptive fields from large and displaced to compact and locally centered and reduces the effective length constant to λ ≈ 200 μm. Together, these data indicate that sparse inhibitory innervation and an excitation-biased E/I ratio enable long-distance integration in TH2 dendrites.

The compartmental model indicated that long electrotonic length constants (λ ≥ 300 μm) are required to generate the observed receptive-field dichotomy. Because synaptic inhibition limits the spread of excitation (*36*–*38*), we investigated the excitation/inhibition (E/I) balance in TH2 dendrites. Comparing TH2 reconstructions with our previous data from VGluT3 (VG3) amacrine cells (which have short λ and amplify object-motion signals in OMS circuits) (*39*, *40*), we found a notable difference in synaptic connectivity. Inhibitory inputs to TH2 dendrites, such as excitatory inputs and outputs (Fig. 2), were distributed uniformly across proximal and distal compartments (proximal: cell 1 = 0.083 and cell 2 = 0.086 syn/μm; distal: 0.066 and 0.075 syn/μm). TH2 dendrites received a twofold higher density of excitatory inputs and half the density of inhibitory inputs compared to VG3 cells, resulting in a high E/I ratio (∼1.30 versus 0.37; Fig. 5, A and B) (*39*).

To test the impact of this synaptic organization, we incorporated inhibitory synapses into our compartmental model. We calibrated membrane conductances and inhibitory currents to yield a physiological λ of 460 μm in the presence of the observed TH2 synaptic densities; under these conditions, the model robustly reproduced the proximal-distal receptive field dichotomy. Replacing the TH2 synaptic densities with those of VG3 cells, while maintaining all other parameters, caused a collapse in simulated signal propagation (λ = 200 μm) and abolished the displaced receptive fields in distal dendrites (Fig. 5, C and D). Thus, our modeling suggests that sparse inhibitory innervation enables the long-distance signal integration required for TH2 wide-field function.

### Voltage-gated sodium channels support long-distance integration in TH2 dendrites

**Fig. 6.**
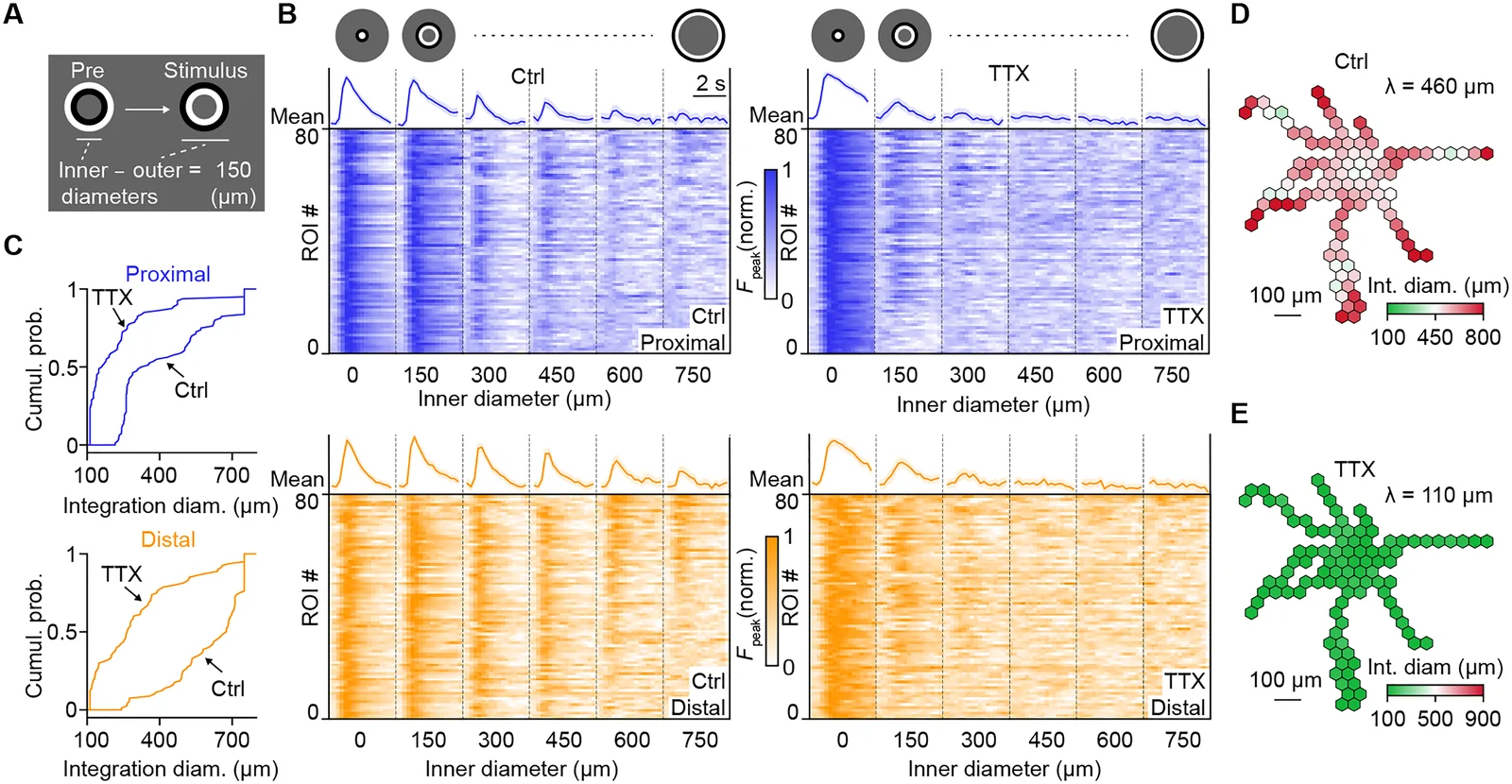
Voltage-gated sodium channels support long-distance integration in TH2 dendrites. (**A**) Concentric contrast-inverting ring stimulus flashed to probe long-distance dendritic integration. Ring width was fixed at 37.5 μm with inner diameters ranging from 0 to 750 μm in 150-μm steps (outer diameter = inner diameter + 150 μm). Each stimulus was presented for 3 s. (**B**) Two-photon calcium responses of the 80 most reliable proximal and distal TH2 ROIs across stimulus sizes under control conditions and in the presence of tetrodotoxin (TTX). For control conditions, *n* = 4 imaging regions from four mice. For TTX, *n* = 4 imaging regions from two mice. Ctrl, control.(**C**) Effective integration diameters for proximal and distal ROIs under control conditions and after TTX. Under control conditions, distal ROIs showed larger integration diameters (607.0 μm [573.9, 640.0 μm]) than proximal ROIs (430.8 μm [387.4, 474.2 μm]). TTX reduced integration diameters in both compartments (distal: 311.8 μm [269.2, 354.5 μm]; proximal: 233.4 μm [195.8, 271.0 μm]). diam., diameters. (**D** and **E**) Compartmental TH2 model shows that adding voltage-gated sodium conductances reproduces effective integration diameters with λ = 460 μm (D). Removing the sodium channels from the modeled cell while maintaining the same passive properties recapitulates the TTX condition with λ = 110 μm (E). Int., integration.

While sparse inhibition facilitates passive propagation, we asked whether active conductances further boost distal signaling (*1*). Although TH2 amacrine cells are nonspiking interneurons (*22*, *23*), we hypothesized that voltage-gated sodium channels might amplify synaptic potentials to support long-distance integration (*41*–*44*). We used a concentric ring stimulus to probe the spatial extent of dendritic integration (Fig. 6A). By varying the inner diameter of the ring (fixed width), we could selectively activate inputs at increasing distances from the recording site. Under control conditions, distal ROIs responded to stimuli with inner diameters up to 600 μm, indicating robust integration of distant signals (Fig. 6B).

Application of tetrodotoxin (TTX) to block voltage-gated sodium channels significantly reduced the integration range. The effective integration diameter (the largest stimulus diameter eliciting >25% of the maximal response) of distal ROIs dropped from 607.0 μm [573.9, 640.0 μm] to 311.8 μm [269.2, 354.5 μm] in the presence of TTX (Fig. 6C). A similar relative reduction from 430.8 μm [387.4, 474.2 μm] to 233.4 μm [195.8, 271.0 μm] was observed in proximal dendrites. To relate these effects to electrotonic parameters, we incorporated voltage-gated sodium channels into the TH2 compartmental model. Constraining the model with the TTX and control data, we found that passive membrane properties alone yielded a λ of 110 μm (simulating TTX; Fig. 6E), whereas the addition of voltage-gated sodium conductances extended λ to 460 μm (simulating control; Fig. 6D). Together, our modeling suggests that sodium channel–mediated amplification synergizes with sparse inhibition to extend the functional reach of distal dendrites.

### Structured distribution of TH2 output across OMS and ON-OFF circuits

**Fig. 7.**
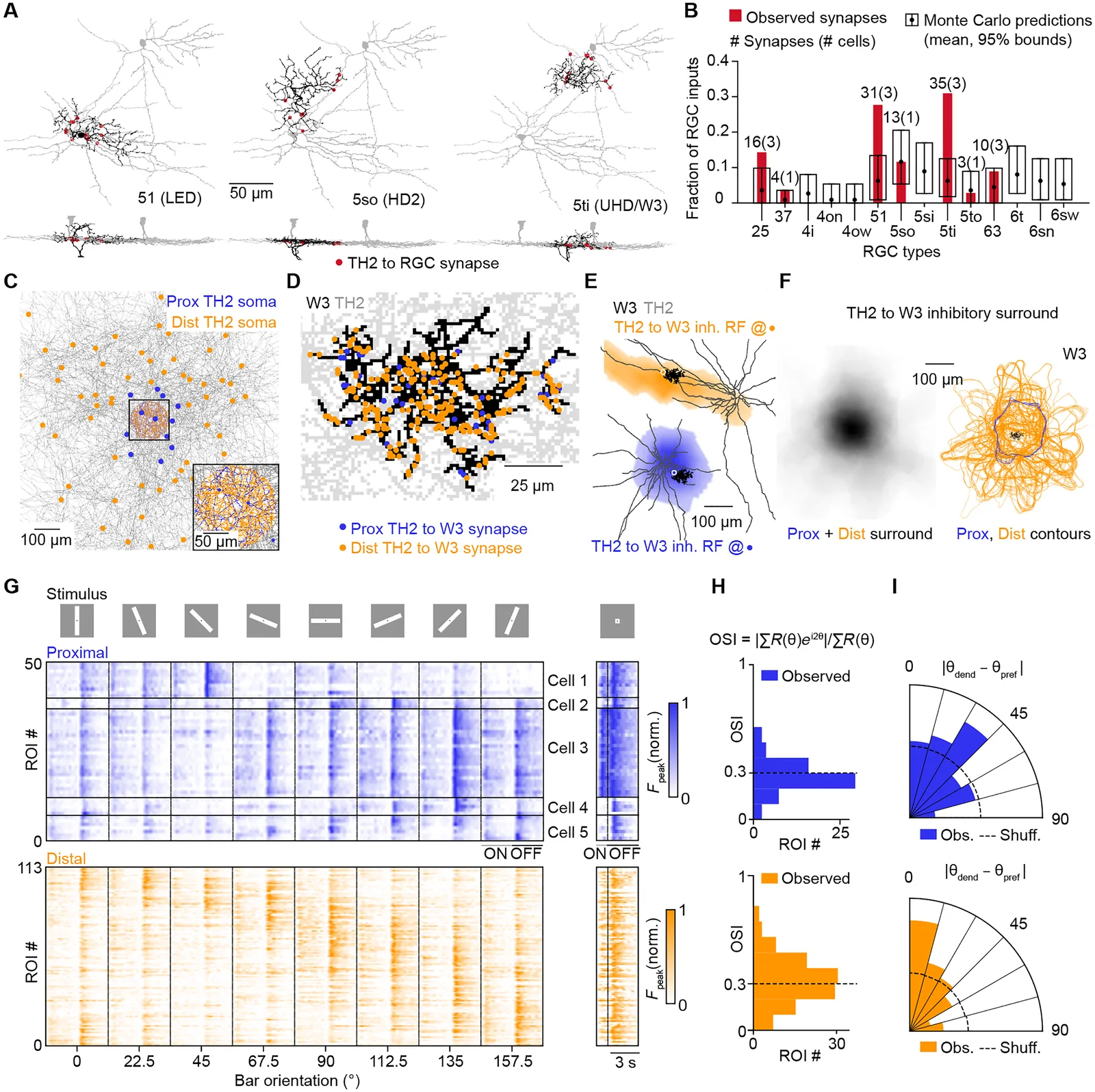
The TH2 output network forms the anatomical basis of geometric filtering. (**A**) Reconstructions of three ganglion cells postsynaptic to TH2 cells, typed by stratification depth and morphology. TH2 cells inhibit three of four OMS types [W3/UHD (5ti), HD2 (5so), and LED (51)] and select non-OMS types [F-mini-ON (63), ON-OFF direction-selective (37), and (25)]. (**B**) Observed fraction of TH2 synapses onto each ganglion cell type versus predictions from dendritic overlap; among OMS cells, a larger fraction targets W3/UHD (5ti) and LED (51). Data from 15 ganglion cells from one retina. (**C**) Population model of the TH2 plexus: One of seven reconstructed arbors was placed at each confocal soma position with random rotation, yielding 196 cells across ∼4 mm^2^. Inset: TH2 dendrites entering a representative 150-μm-diameter region (proximal: blue; distal: orange). (**D**) A reconstructed W3/UHD cell embedded in the plexus received 38.5 synapses from 3.4 proximal and 209.2 synapses from 38.6 distal TH2 cells (40 iterations). (**E**) Inhibitory receptive fields at single TH2-to-W3/UHD synapses: Proximal synapses mediate compact, locally centered suppression; distal synapses mediate large, displaced suppression oriented along the presynaptic dendrite. inh., inhibitory. (**F**) Left: Summed TH2 inhibition onto a W3/UHD cell, a dense proximal core within a diffuse distal halo. Right: Proximal (blue) and distal (orange) contours reveal a circular near-surround with radially oriented far-surround subunits (daisy shape). Thresholds in (E) and (F) are 25% of max. (**G**) Two-photon imaging of TH2 dendrites during oriented bar stimulation (800 × 50 μm, 0° to 180° in 22.5° steps), with ROIs sorted by preferred orientation (*n* = 5 imaging regions from three mice). (**H**) Orientation selectivity index (OSI) histograms; ROIs with OSI > 0.3 were classified as tuned, with more in distal (54.9%) than proximal (36.0%) dendrites. (**I**) Distal ROIs showed stronger alignment between local dendrite and preferred response orientation (33.1°) than shuffled (Shuff.) data (45.0°). Obs., observed.

Having identified the biophysical mechanisms that allow individual TH2 arbors to multiplex local and global signals, we next sought to understand how these single-cell properties scale to shape the computational geometry of downstream circuits. To map TH2 output distribution, we reconstructed 15 postsynaptic ganglion cells from our 3DEM dataset and assigned each to a type based on stratification depth and similarity to EyeWire reconstructions (Fig. 7, A and B) (*45*). TH2 amacrine cells provided inhibition to three of the four known OMS ganglion cell types (*46*): W3/UHD (EyeWire 5ti) (*22*, *29*), High-Definition-2 (HD2) (5so) (*47*), and Local Edge Detector (LED) (51; Fig. 7A). In each case, proximal and distal TH2 compartments contributed convergent output. TH2 cells also contacted F-mini-ON (63), ON-OFF direction-selective (37), and a physiologically uncharacterized type (25; fig. S6).

To analyze the specificity of these connections, we compared the observed fraction of TH2 synapses onto each ganglion cell type to predictions based solely on dendritic overlap between TH2 arbors and all ganglion cells in the EyeWire dataset (Fig. 7B). Among OMS cells, W3/UHD and LED received substantially more TH2 input than expected from overlap alone, whereas HD2 matched predictions and HD1 (5si) received no input despite comparable expected innervation. Among non-OMS targets, type 25 was preferentially innervated, while ON-OFF direction-selective and F-mini-ON [an ON-OFF responsive type despite its name (*48*)] received inputs within the predicted range; several costratifying transient OFF (4i, 4on, and 4ow/tOFFα) and transient ON types (6 t, 6sn, and 6sw) received no synapses despite substantial overlap (Fig. 7B). Thus, TH2 output is highly structured: These cells preferentially innervate ganglion cells that combine ON and OFF signals, well suited for pattern- and contrast-independent motion sensing (*49*, *50*), while avoiding transient unipolar ON and OFF pathways.

### The complex structure of TH2-mediated surrounds of W3/UHD ganglion cells

Having established the biophysical rules governing single-cell integration, we next asked how these rules scale to determine the geometric properties of the population-level neuropil. Because W3/UHD ganglion cells emerged as dominant TH2 targets, we focused on them to ask how the dense TH2 plexus constructs inhibitory surrounds. We built a population model by extracting soma positions from confocal maps and filling each with a randomly rotated TH2 arbor from our seven reconstructions, yielding 196 cells across ∼4 mm^2^ (Fig. 7C). A reconstructed W3/UHD cell was embedded in this plexus, and synapses were assigned probabilistically wherever TH2 and W3/UHD dendrites came within 1 μm, with connection probability calibrated to match our 3DEM observation of ∼10 synapses per proximal TH2 arbor. Repeating this circuit generation 40 times yielded consistent connectivity patterns: Each W3/UHD cell received 38.5 [37.5, 40.0] synapses from 3.4 [3.1, 3.7] proximally connected TH2 cells and 209.2 [179.7, 238.6] synapses from 38.6 [36.5, 40.7] distally connected cells (Fig. 7D), an approximately sixfold bias toward distal input that matches the greater length of distal dendrites (Fig. 1G).

To determine the spatial structure of the resulting surround, we mapped the receptive field of each synaptic contact using compartmental simulations (fig. S4). Synapses from proximal compartments contributed compact, locally centered inhibitory receptive fields (Fig. 7E), whereas synapses from distal compartments contributed large, spatially offset fields aligned with the orientation of the presynaptic dendrite (Fig. 7F). Summing across all synapses predicted a daisy-shaped composite surround: a circular near-surround driven by proximal inputs embedded within a halo formed by radially oriented distal receptive fields (Fig. 7G). Thus, the bipartite TH2 morphology is predicted to construct a complex surround that combines strong local suppression with broad, orientation-specific signal integration.

### Distal TH2 dendrites exhibit orientation-tuned receptive fields aligned with arbor geometry

The radially oriented far-surround subunits in our population model and the elongated distal receptive fields in our single-cell model predict that distal TH2 dendrites should be driven preferentially by stimuli aligned with their anatomical orientation (*51*, *52*), whereas proximal dendrites should not (Fig. 7, E to G). To test these predictions, we imaged calcium responses to oriented bars (800 × 50 μm; 3-s ON, 3-s OFF; Fig. 7H) and computed an orientation selectivity index (OSI) from the vector sum of responses (Fig. 7I). Distal ROIs were more orientation selective than proximal ROIs: 54.9% versus 36.0% exceeded a threshold of OSI > 0.3 (mean difference in OSI: 0.043 [0.005, 0.081]).

We next asked whether the orientation preferences of distal ROIs align with their dendritic orientation. Comparing each ROI’s preferred stimulus orientation (θ_pref_) with the local dendritic orientation (θ_dend_), we quantified the alignment as |θ_pref_ – θ_dend_| (Fig. 7J). Distal response preferences were closely aligned with the dendritic axis (mean difference of 33.1° [28.4, 37.8] versus 45.0° [40.2, 49.8] for shuffled data). Proximal ROIs, by contrast, showed orientation preferences clustered by cell identity rather than local dendritic geometry. Thus, distal TH2 dendrites form orientation-tuned subunits whose preferred axes follow the underlying dendritic architecture.

### Orientation-tuned far-surround subunits silence W3/UHD ganglion cells

**Fig. 8.**
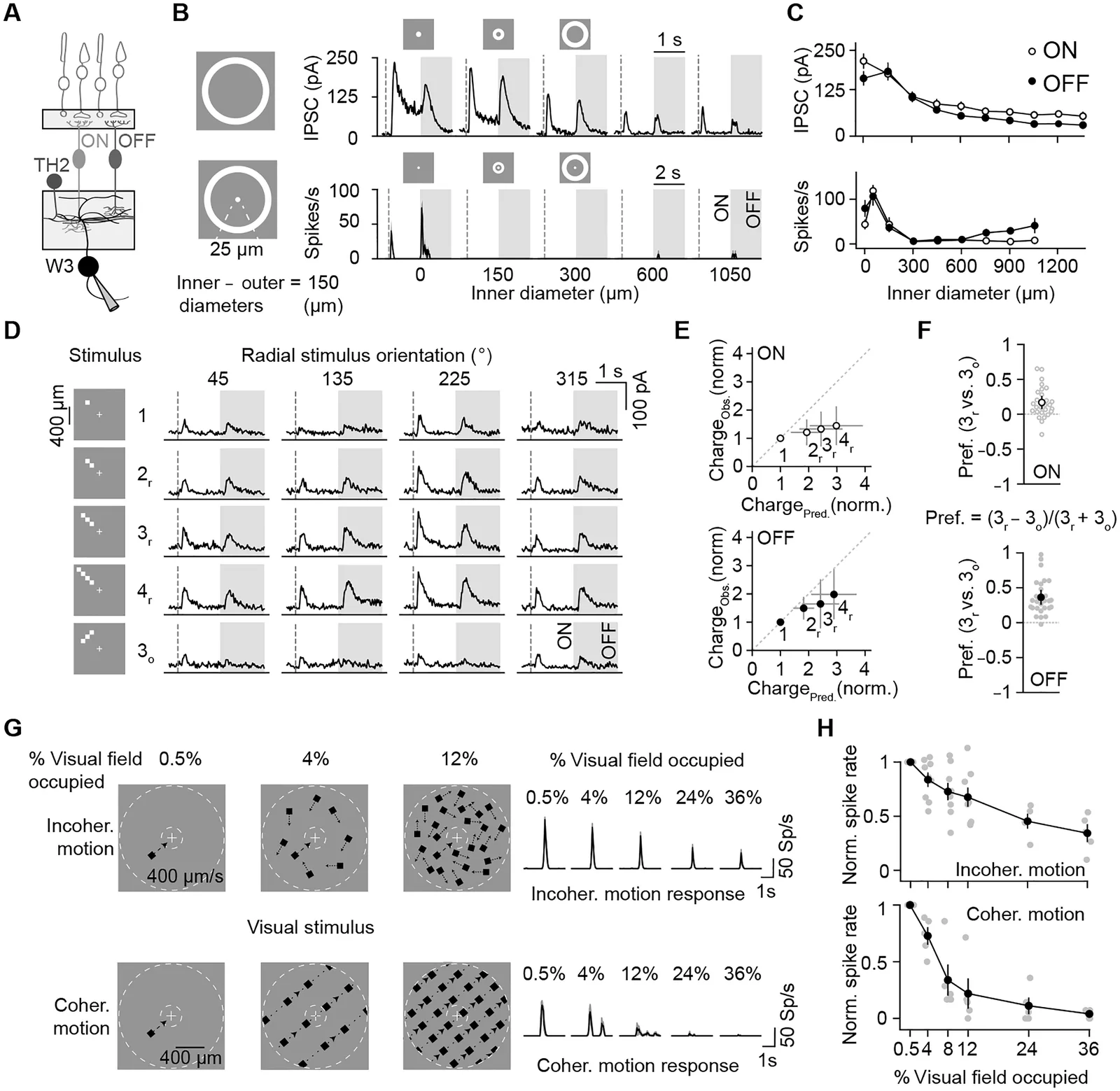
Radially orientation-selective far-surround subunits silence W3/UHD ganglion cells during global motion while sparing object responses in clutter. (**A**) Circuit schematic: TH2 amacrine cells inhibit W3/UHD ganglion cells. (**B**) Example W3/UHD recordings. Top: Inhibitory postsynaptic currents (IPSCs) evoked by contrast-reversing annuli of increasing inner diameter (3-s ON, 3-s OFF). Bottom: Spike responses to a 25-μm central spot alone or with surrounding annuli. (**C**) Top: Peak IPSC amplitude versus annulus inner diameter (*n* = 7 cells from three mice). Bottom: Peak spike rate evoked by the central spot with surrounding annuli (*n* = 10 cells from four mice). (**D**) Left: Stimulus testing radial organization, with 100-μm squares along radial axes (45°, 135°, 225°, and 315°) or arranged orthogonally. Right: Example IPSCs. (**E**) Sublinear spatial summation within radial subunits: observed versus predicted inhibitory charge for one to four radially arranged squares (ON and OFF; *n* = 7 cells from three mice, four angles per cell). (**F**) Inhibitory charge (normalized to the single proximal square) for three squares arranged radially (3r) versus orthogonally (3o). Radial configurations evoke stronger OFF inhibition [Radial Preference Index (Pref. or RPI): 0.363 [0.266, 0.460]; *n* = 7 cells from three mice]. (**G**) Testing motion-coherence selectivity. Top: “Probe + clutter” stimuli with increasing densities of incoherent moving objects (0.5 to 36% visual field occupancy). Bottom: Coherent global grid motion. Traces show W3/UHD spike responses to the central probe, robust across incoherent densities but suppressed by coherent motion from 12% occupancy. Sp/s, spikes/second; Incoher., incoherent; Coher., coherent. (**H**) Normalized maximum spike rates for incoherent (*n* = 9 cells from three mice) and coherent (*n* = 5 cells from two mice) motion, normalized to each cell’s response to the probe alone (0.5% occupancy). W3/UHD responses are resilient to incoherent object motion but suppressed by coherent grid motion, showing that the TH2-mediated surround filters global self-motion while preserving object detection in clutter.

Our population model predicts that W3/UHD ganglion cells receive significant inhibition from stimuli far beyond their dendritic field and that this far-surround is composed of radially oriented subunits (i.e., the petals of the daisy-shaped surround). We patch clamped W3/UHD ganglion cells and presented rings of increasing inner diameter (3-s ON, 3-s OFF). Cells received robust inhibition over a large spatial range, with substantial inhibitory postsynaptic currents (IPSCs) even for annuli with inner diameters of 1050 μm, nearly 10 times the ∼115-μm dendritic field diameter of W3/UHD cells (Fig. 8, A to C) (*20*, *53*, *54*). Although the local circular contour of an annulus is orthogonal to the axis of any individual radial subunit, flashing this stimulus synchronously across the entire 360° visual field recruits the full halo of far-surround dendrites simultaneously; this spatial summation overcomes local orientation preferences to drive strong aggregate inhibition. In paired stimuli, these annuli, including the largest, effectively suppressed the spiking evoked by a 25-μm central spot (Fig. 8, B and C).

To test for radial organization, we measured IPSCs evoked by 100-μm squares positioned along radial axes (45°, 135°, 225°, and 315°) or arranged orthogonally (Fig. 8D). Single squares and radially arranged multiples evoked robust IPSCs along all axes, with multiple squares summing sublinearly (Fig. 8E). Critically, three squares arranged radially evoked OFF IPSCs approximately twice as large as three squares arranged orthogonally, whether normalized to a shared single-square baseline (Fig. 8, D and F) or compared to their linear-sum predictions (fig. S7), confirming radial orientation selectivity of the far-surround subunits independent of absolute square positions.

### Daisy-shaped surround geometry generates motion-coherence selectivity

Having confirmed that the W3/UHD inhibitory surround extends far beyond the dendritic field and that the far-surround is composed of radially orientation-selective subunits, we next asked what visual computation this geometry implements. Broad surround inhibition is necessary to suppress coherent global motion during self-motion, but a uniform broad surround would risk silencing W3/UHD responses whenever many objects move in the surround. We hypothesized that the daisy-shaped geometry resolves this trade-off by transforming motion coherence into differential inhibition.

Coherent global motion, such as optic flow, sweeps uniformly across the visual field. Because the far-surround subunits provide complete radial coverage, any uniform translational motion will inevitably align with the preferred axis of a subset of these subunits (on opposite sides of the receptive field), synchronously driving the strong inhibition required to cancel the global signal. In contrast, in a cluttered scene, objects move independently, and most will travel along nonradial trajectories relative to the receptive field center. The radial orientation selectivity of the far-surround subunits mitigates oversuppression by geometrically filtering out these nonradial trajectories. Even when individual objects do align with a preferred radial axis, they do so at uncorrelated times, resulting in weak, asynchronous inhibition.

To test this prediction, we recorded W3/UHD spiking while presenting a central probe object together with surrounding motion at matched densities (Fig. 8, G and H, and movie S1). Under the incoherent condition, surrounding dark squares moved independently, while the probe traversed the receptive-field center. W3/UHD responses to the probe remained robust, as visual-field occupancy increased from 0.5 to 36%. Under the coherent condition, identical dark squares translated together as a global grid, matching element size, speed, arena, and occupancy. Unlike dense incoherent motion, coherent motion strongly suppressed W3/UHD spiking at matched densities (Fig. 8, G and H). Thus, W3/UHD cells are not simply selective for small objects over large (fig. S7) or dense stimuli. Instead, the TH2-mediated daisy-shaped surround generates motion-coherence selectivity, suppressing coherent self-generated image motion while preserving object responses during dense incoherent motion.

## DISCUSSION

Our study establishes a comprehensive, multiscale framework for how single-cell structural and biophysical specializations scale to construct complex, population-level algorithmic filters. Using the retina as a model, we decipher the construction of inhibitory surrounds, which are critical for sensory feature detection. (i) We show that TH2 amacrine cells have a bipartite morphology, comprising a dense proximal arbor and a sparse, radiating distal array. (ii) Using CLEM, we find that input and output synapses are distributed uniformly across this bipartite structure, challenging the notion that functional segregation requires synaptic asymmetry. (iii) We demonstrate that despite this synaptic uniformity, TH2 cells generate compartmentalized receptive fields: proximal dendrites perform local processing, while distal dendrites integrate signals over hundreds of micrometers. (iv) We identify the biophysical mechanisms (sparse inhibition and voltage-gated sodium channels) that tune the electrotonic length constant to support this long-distance propagation without generating spikes. (v) By embedding these biophysical properties into a population model, we show how the TH2 plexus constructs a specific inhibitory landscape for postsynaptic targets, comprising a circular near-surround and a far-surround composed of radially oriented subunits. (vi) We demonstrate that geometric filtering in this daisy-shaped composite surround generates motion-coherence selectivity, allowing W3/UHD ganglion cells to distinguish global self-motion from the incoherent motion of multiple objects, resolving a fundamental trade-off in object-motion sensitivity.

In many retinal interneurons, functional compartmentalization relies on the asymmetric distribution of inputs and outputs (*4*, *9*, *13*, *55*, *56*). For example, AII amacrine cells segregate inputs and outputs to distinct neurite lobules to mediate crossover inhibition (*57*, *58*), and starburst amacrine cells restrict release sites to the distal tips of their dendrites to generate direction selectivity (*9*, *59*, *60*). In contrast, TH2 amacrine cells achieve compartmentalization through a distinct strategy: a bipartite branching pattern combined with uniform synaptic density. Our modeling suggests that this morphological strategy relies on tuning the electrotonic length constant (λ) to a specific window. If λ is too short, as seen in A17 or VG3 amacrine cells (*39*, *40*, *61*, *62*), the neuron functions as a collection of isolated local processing units. If λ is too long or supported by regenerative spiking, as in dopaminergic or primate polyaxonal amacrine cells (*30*, *63*–*65*), the arbor becomes a global, monolithic integrator. Our modeling suggests that TH2 cells occupy a critical intermediate regime where λ allows signal propagation along unbranched distal neurites while permitting charge dissipation in the complex proximal arbor. This architectural logic appears to be a variation on a theme found in other wide-field interneurons; for instance, sustained monopolar wide-field amacrine cells with orientation-selective dendrites connect to sustained retinal ganglion cells (RGCs) but lack the dense proximal compartment of the TH2 (*51*, *52*). This suggests that the retina tunes the electrotonic properties of amacrine cell neurites to translate diverse branching patterns into specific computational architectures tailored to distinct circuit functions.

The capacity of TH2 distal dendrites to integrate signals over large distances relies on specific biophysical mechanisms that distinguish them from locally processing amacrine cells. We found that TH2 dendrites operate with a high E/I ratio (>1), receiving sparse inhibition relative to excitation. This contrasts sharply with VG3 amacrine cells, where strong inhibition (E/I ≪ 1) shunts charge and confines integration to narrow dendritic domains (*39*, *40*, *61*). Furthermore, we identified a critical role for voltage-gated sodium channels. Unlike dopaminergic amacrine cells and polyaxonal amacrine cells in primates that use sodium channels to drive action potentials for global signal broadcast (*30*, *63*–*65*), TH2 cells appear to use these channels to boost synaptic potential without eliciting spikes. The absence of spiking, despite the presence of voltage-gated sodium channels, likely results from channel densities tuned to balance amplification against instability (*66*). Although TTX acts globally, bipolar-cell–mediated responses to small local stimuli remained intact under our conditions (Fig. 6B), and analogous protocols have been used to isolate amacrine contributions in OMS and related circuits while preserving feedforward drive (*67*), supporting a TH2-dendritic origin of the integration deficits. It remains an open question whether these integration properties are static or adaptable; given that starburst amacrine cells can adjust their responses to stimulus conditions (*68*, *69*), it will be interesting to determine whether TH2 cells similarly modulate their excitability or synaptic weights to adapt the spatial extent of the surround.

While the properties of single amacrine cells inform us about subcellular computation, understanding their impact on the visual code requires analyzing the collective function of the amacrine cell plexus. By transitioning from single-cell reconstructions to a population framework, our model predicted a counterintuitive structure for the W3/UHD inhibitory surround: a dense proximal plexus creating a circular near surround, embedded within a halo of distal neurites creating radial subunits of the far surround. The prediction that the far-surround is composed of radially orientation-selective subunits was confirmed by our two-photon calcium imaging and patch-clamp recordings. This correspondence between the population model and physiological data underscores a principle applicable across brain regions: We must analyze interneuron function at the level of the circuit neuropil (*70*). The geometry of the plexus itself is a primary computational variable, determining how the output of individual neurons converges to shape the receptive fields of downstream targets.

The functional signature of the TH2 plexus is directed with high specificity toward transient ON-OFF ganglion cells while avoiding costratifying transient unipolar ON or OFF types. This target selectivity suggests that the complex surround mediated by TH2 cells is specialized for circuits that encode motion in a contrast- and pattern-invariant manner (*49*, *50*). The inclusion of targets beyond canonical OMS types, such as type 25 and F-mini-ON (*48*), raises the possibility that object-motion encoding extends more broadly across the transient ON-OFF ganglion cell population than currently appreciated (*19*). While global motion suppression is a necessary computational theme across all OMS ganglion cell types, these cells exhibit distinct speed and size tuning preferences. We found that TH2 acts as a common denominator within these diverse circuit motifs, providing shared inhibition across multiple OMS types. However, the strength of this innervation varies significantly across targets (from dense inputs to W3/UHD and LED cells to sparse contacts on others), suggesting that the TH2 plexus provides differentially weighted inhibition, allowing each ganglion cell type to encode distinct variations on a common computational theme.

The discovery of the daisy-shaped surround forces a reevaluation of the fundamental computation performed by OMS circuits. While OMS cells are classically described by their suppression to large or dense stimuli, this size-based description obscures the underlying algorithmic logic (*17*, *19*, *22*, *54*). The specific geometry of the TH2 plexus reveals that the circuit is built not simply to measure stimulus size or density but to solve a more complex naturalistic problem: separating coherent self-motion from incoherent object motion in cluttered environments. Our data reveal that the TH2 far surround solves this problem through geometric filtering. Because the far-surround subunits provide complete radial coverage, wide-field coherent translational motion aligns with the preferred axis of a subset of petals (those parallel to the motion trajectory) and recruits them synchronously, driving the strong inhibition required to cancel the global signal. In contrast, in a cluttered scene of independently moving objects, most objects travel along nonradial trajectories relative to the W3/UHD center, which are geometrically filtered out. Even when individual objects align with a preferred radial axis, they do so at uncorrelated times, resulting in weak, asynchronous inhibition. By filtering out nonradial trajectories and asynchronous signals, this geometric architecture spares W3/UHD ganglion cells from overwhelming suppression, allowing them to robustly encode object motion even as the density of surrounding movement increases. Thus, the bipartite morphology of the TH2 cell serves as a highly specific geometric adaptation, converting a dense anatomical plexus into a precise spatial filter for motion coherence.

Our approach has certain limitations. First, our 3DEM volume captured a fraction of the expansive TH2 arbors, requiring extrapolation across the plexus. Second, the precise subcellular distribution of sodium channels in our model remains an estimate. Last, our population model assumes static connectivity, while biological surrounds may be dynamically modulated (*71*). For these principles to be applied broadly, future studies must determine whether analogous morphological specializations construct geometric filters in other dense neuropils, such as the cerebral cortex. In addition, abstracting the algorithmic logic of the TH2 plexus could directly inform neuromorphic engineering, providing artificial vision systems with spatial filters that robustly segregate self-motion from object motion. By linking single-cell form to population-level algorithmic logic, this architecture resolves a fundamental sensory trade-off, ensuring robust signal detection within the dynamic clutter of the natural world.

## MATERIALS AND METHODS

### Animals

Throughout this study, we used combinations of the following mouse strains: TH-Cre (*27*) (The Jackson Laboratory, stock #008601), Ai9 (*72*) (Cre-dependent tdTomato reporter; The Jackson Laboratory, stock #007909), and Ai148 (*73*) (Cre-dependent GCaMP6f calcium indicator; The Jackson Laboratory, stock #030328). TH2 amacrine cells were labeled for correlative light and electron microscopy (CLEM) by crossing TH-Cre to Ai9 and for two-photon calcium imaging by crossing TH-Cre to Ai148. Wild-type C57BL/6J mice were used for patch-clamp electrophysiology. All transgenic lines were maintained on a C57BL/6J background and bred in-house. Mice were housed on a 12-hour light/dark cycle with food and water available ad libitum. Retinas were isolated from mice of both sexes between postnatal day 30 (P30) and 4 months of age. All procedures were approved by the Institutional Animal Care and Use Committee of Washington University School of Medicine (Protocol # 23-0116) and performed in accordance with the National Institutes of Health Guide for the Care and Use of Laboratory Animals.

### Viral constructs and intraocular injections

To sparsely label TH2 amacrine cells, TH-Cre pups (P6) were briefly anesthetized and injected intraocularly with 350 nl of a 1:1 mixture of AAV-EF1a-BbChT (AAV9; Addgene viral prep #45186-AAV9) and AAV-EF1a-BbTagBY (AAV9; Addgene viral prep #45185-AAV9). These vectors encode Brainbow fluorophores under the elongation factor-1 alpha (EF1α) promoter (*28*). Injections were delivered into the vitreous of each eye using a Nanoject II injector (Drummond Scientific). Retinas were dissected 4 to 8 weeks after injection to allow stable expression of Brainbow fluorophores for confocal imaging.

### Tissue preparation

Mice were dark adapted for more than 1 hour, deeply anesthetized with CO_2_, euthanized by cervical dislocation, and enucleated under infrared illumination. Retinas were isolated in mouse artificial cerebrospinal fluid buffered either with Hepes (mACSF_Hepes_; used for confocal and correlative light and electron microscopy) or sodium bicarbonate (mACSF_NaHCO3_; used for two-photon imaging). mACSF_Hepes_ contained 119 mM NaCl, 2.5 mM KCl, 2.5 mM CaCl_2_, 1.3 mM MgCl_2_, 1 mM NaH_2_PO_4_, 11 mM glucose, and 20 mM Hepes (pH adjusted to 7.37 with NaOH). mACSF_NaHCO3_ contained 125 mM NaCl, 2.5 mM KCl, 1 mM MgCl_2_, 1.25 mM NaH_2_PO_4_, 2 mM CaCl_2_, 20 mM glucose, 26 mM NaHCO_3_, and 0.5 mM l-glutamine equilibrated with 95% O_2_/5% CO_2_.

### Confocal imaging

For confocal imaging of TH2 amacrine cells, retinas from Brainbow-labeled TH-Cre mice were fixed in 4% paraformaldehyde in phosphate-buffered saline (PBS) for 30 to 45 min at room temperature, washed in PBS, and flat mounted ganglion cell side up on black membrane disks (HABGO1300, MilliporeSigma). Whole mounts were imaged on an Olympus FV1000 laser-scanning confocal microscope using 20× and 40× oil-immersion objectives. For Brainbow experiments, we identified sparsely labeled TH2 cells by visually isolating individual multicolor arbors in low-magnification overview stacks and then acquired high-magnification z-stacks spanning the inner nuclear to ganglion cell layers (GCLs). For both magnifications, we used tiled acquisitions to cover the full extent of each TH2 arbor.

For correlative light and electron microscopy, retinas from TH-Cre; Ai9 mice were fixed in 2% paraformaldehyde and 2% glutaraldehyde, stained with 4′,6-diamidino-2-phenylindole (DAPI), and imaged on the same confocal setup. We acquired multiresolution z-stacks of the labeled region, capturing DAPI-labeled nuclear architecture in the ganglion cell and inner nuclear layers (INLs), retinal vasculature, and tdTomato-labeled TH2 cells in the INL. TH-Cre; Ai9 sparsely labels dopaminergic amacrine cells (*22*, *74*). TH2 cells were distinguished from dopaminergic amacrine cells by the stratification depth of their dendrites in the IPL. These confocal maps provided fiduciary landmarks that were later registered to the multiresolution 3DEM dataset, enabling relocation of the same TH2 cells for ultrastructural reconstruction and synapse annotation (*33*).

### Morphological reconstruction of TH2 cells from confocal imaging

To quantify dendritic morphology in Brainbow-labeled retinas, individual TH2 amacrine cells were segmented and skeletonized from high-magnification confocal z-stacks. We reconstructed seven complete TH2 arbors from four mice for morphological analysis. Confocal volumes were exported as TIFF stacks and loaded into napari (*75*). We used the 3D stack to resolve ambiguous branch patterns, but skeletonization was performed in 2D within the narrow TH2 stratification band. For each cell, we segmented the arbor and skeletonized branches from the soma to the tips. The resulting skeleton graph was saved as an SWC file, a standard format for neuronal morphologies (*76*). Custom Python scripts were used to implement the napari-based skeletonization workflow and to extract path lengths, interbranch distances, and compartment labels for analysis. Dendrites were classified as proximal or distal using the local interbranch distance criterion established in Fig. 1 (44.1 μm), which is independent of soma position and can therefore be applied uniformly across light microscopy, electron microscopy, and two-photon imaging fields where parent-soma assignment for individual neurites is not tractable.

### Electron microscopy

For 3DEM, retinas were initially fixed in 2% paraformaldehyde and 2% glutaraldehyde in 0.1 M cacodylate buffer and imaged for the confocal CLEM pipeline as described above. Following imaging, retinal pieces containing TH2 somata were trimmed on the basis of the confocal maps and postfixed for electron microscopy. Tissue was rinsed in 0.1 M cacodylate and processed with a heavy-metal en bloc protocol adapted from (*77*) and our multiresolution CLEM workflow (*33*). Briefly, samples were incubated in osmium tetroxide, reduced osmium with potassium ferrocyanide, thiocarbohydrazide/pyrogallol (osmium linking), a second osmium tetroxide incubation, aqueous uranyl acetate, and lead aspartate, followed by graded organic solvent dehydration and embedding in epoxy resin.

Ultrathin sections (40 nm) were cut with an ultramicrotome equipped with a diamond knife and collected on Kapton tape using an automated tape-collecting ultramicrotome (ATUMtome, RMC Boeckeler) and then mounted on silicon wafers for imaging in a Zeiss Merlin field-emission scanning electron microscope with a backscatter detector. We use WaferMapper software (*78*) to acquire low- and medium-resolution overview mosaics spanning a ∼300 × 300 μm region from GCL to INL to identify the retinal area containing TH2 cell bodies. Within this volume, the central IPL band where TH2 dendrites stratify was imaged at high resolution (4 × 4 × 40 nm voxels), while surrounding regions were acquired at lower in-plane resolution (8 × 8 × 40 nm), yielding a ∼300 × 300 × 48 μm multiresolution 3DEM dataset.

To locate the same TH2 cells identified in confocal maps, 3DEM overview images were registered to the fixed confocal mosaics using blood vessels, retinal layer boundaries, and DAPI-labeled nuclear patterns as fiducial landmarks. We then matched tdTomato-positive somata in the INL with dendrites stratifying at mid-IPL depth (TH2 cells) between confocal and 3DEM datasets and traced these neurons through the high-resolution stack for subsequent ultrastructural reconstruction and synapse annotation.

### 3DEM segmentation and skeletonization

The 3DEM volume was aligned by ScalableMinds. TH2 amacrine cells and their synaptic partners were manually segmented from the high-resolution 3DEM volume in volume annotation and segmentation tool (*79*). Cell bodies, dendrites, and axon terminals were traced across consecutive sections to generate filled 3D segments, and putative synapses were annotated as membrane specializations with clustered presynaptic vesicles and a defined synaptic cleft; ribbon synapses were additionally marked by the presence of an electron-dense ribbon. For each TH2 cell, the segmented volume and associated synapse annotations were imported into CellNav (https://github.com/MorganLabShare/CellNav), where we generated skeleton representations, verified reconstruction continuity, assigned synapses to individual TH2 arbors, and extracted path lengths, branch points, and synapse positions along proximal and distal compartments for subsequent quantitative analyses. Because TH2 arbors stratify at the border of the ON and OFF plexuses (∼50% IPL depth), we distinguished ON from OFF bipolar inputs by partially reconstructing presynaptic bipolar axons to determine whether they stratify in the ON or OFF sublamina.

### Monte Carlo analysis of cell type connection probability

Depth-distribution profiles for bipolar and ganglion cell types were downloaded from the EyeWire Museum (*45*). To align our IPL coordinate system with EyeWire, we computed a depth correction factor so that the stratification of reference bipolar and ganglion cell types in our 3DEM dataset matched their reported IPL depths. For each ganglion cell type, we normalized the total IPL arbor within the volume to equalize it across types, compensating for the underrepresentation of wide-field cells. Using skeletons of reconstructed TH2 arbors, we then computed a depth-overlap profile between TH2 dendrites and each ganglion cell type by multiplying their depth distributions and integrating over IPL depth. In the Monte Carlo model, the anatomically identified TH2-to-ganglion cell synapses in our dataset were reassigned 10,000 times with probabilities defined by these depth-overlap weights, yielding a depth-constrained null distribution of synapse counts for each partner type. Confidence intervals reported in the text correspond to central quantiles of the ranked Monte Carlo outcomes (for example, the 0.5 to 99.5% percentiles for 99% intervals)

### Two-photon calcium imaging

Two-photon calcium imaging was performed on a custom-built upright microscope (HyperScope, Scientifica) controlled by ScanImage r3.8 (MATLAB) and a PCI-6110 data acquisition board (National Instruments). GCaMP6f was expressed in TH2 amacrine cells via the TH-Cre; Ai148 cross and excited with an InSight DS+ laser (Spectra-Physics) tuned to 930 nm. Laser intensity at the sample was maintained below 3.5 mW. Emitted fluorescence was collected through a 20×, 1.0 numerical aperture water-immersion objective (Olympus), passed through a 450-nm long-pass filter (Thorlabs), and isolated with a 513- to 528-nm band-pass filter (Chroma) before detection by a photomultiplier tube.

Retinas from dark-adapted (>1 hour) mice were dissected under infrared illumination in mACSF_NaHCO3_, flat mounted on transparent membrane disks (Anodisc 13, Cytiva), and perfused (3 to 7 ml/min) with warm (31° to 33°C) mACSF_NaHCO3_ equilibrated with 95% O_2_/5% CO_2_ throughout imaging. Images were acquired at 8.46 or 16.67 frames/s with a spatial sampling of 21.64 or 5.43 pixels/μm, respectively. Imaging planes were positioned at the TH2 stratification depth in the middle IPL. For each field, retinas were allowed to adapt to the laser for 30 s before visual stimulation. Image series with detectable z-drift were excluded on the basis of simultaneously acquired transmitted-light images and subsequent rigid registration.

### Pharmacology

For experiments involving sodium channel blockade, TTX citrate (Alomone Labs) was added to the perfusion solution at a final concentration of 0.5 μM. Retinas were perfused with TTX for 15 min before recording to ensure complete blockade of sodium channels. This concentration and incubation time were chosen to ensure complete block of voltage-gated sodium channels throughout the IPL, including along the long, unbranched distal TH2 dendrites (>500 μm from the soma), where drug equilibration in the whole-mount preparation is slower than at superficial somatic membranes (*67*).

### Visual stimulation—Two-photon imaging

Visual stimuli were generated in MATLAB using Cogent graphics and presented with a fiber-optic coupled DLP projector (E4500 MKII PLUS II, EKB Technologies) illuminated by a 385-nm light-emitting diode (LED; Thorlabs) and focused onto the photoreceptor layer through the substage condenser (*40*). Projector output was attenuated with neutral density filters and calibrated with a spectrometer (BLACK-Comet, StellarNet) and a photometer (S450, UDT) to achieve mean intensities of 8600 isomerizations per rod per second (R*) and 1800 isomerization per S-cone per second (S*). To prevent contamination of the fluorescence signal and to restrict stimulation to galvo flyback, we used a custom open-source Printed Circuit Board (PCB) based on the LCr add-on board from the Open Visual Stimulator project (*80*) to gate the projector LEDs with the scan-line blanking signal. An Arduino microcontroller generated Transistor-Transistor Logic (TTL) pulses that were recorded as a digital input on channel 4 of the ScanImage acquisition board and used to synchronize stimulus timing with the imaging data. Stimuli were written in MATLAB using Cogent graphics extensions (J. Romaya, University College London, London, UK).

### Calcium imaging analysis

Calcium imaging data were analyzed in MATLAB and Python. Time series were first inspected for z-drift using the transmitted-light channel; recordings with detectable z-displacements were discarded. For retained fields, frames were rigidly registered to the median frame based on transmitted-light images, and the resulting transformations were applied to the GCaMP6f channel. ROIs were segmented on the TH2 plexus using custom Python scripts in napari (*75*) at the TH2 stratification depth and were manually inspected and refined. ROIs were classified as proximal or distal using the interbranch distance criterion described above. For receptive-field mapping experiments, in which the stimulus varied along a single horizontal axis, distal ROIs were further classified by local dendritic orientation: ROIs whose dendritic axis fell within ±45° of horizontal were included in the main analyses (Fig. 3); the remaining vertically aligned distal ROIs were analyzed separately (fig. S3).

For each ROI, we computed the relative fluorescence change as

$$\frac{∆F}{F}=\frac{\left[F\left(t\right)-F_{0}\right]}{F_{0}}$$

where *F*_0_ was the median fluorescence during a baseline window preceding stimulus onset, pooled across repeats. Trial-averaged response traces were obtained for each stimulus condition.

### Receptive field and integration analysis

To map receptive fields, we presented a vertically oriented bar of positive contrast (height: 80 μm, width: 50 μm; 3-s ON and 3-s OFF) on a gray background at positions ranging from −300 μm to +300 μm along the horizontal axis relative to the center of the imaging region (27.5 μm high, 55 μm wide). Bar positions were interleaved in pseudorandom order and repeated several times with gray intervals between flashes. For each bar position and polarity, we extracted the peak Δ*F*/*F* in a fixed poststimulus window and averaged across repeats to obtain spatial response profiles. Because OFF responses dominated, receptive field analyses reported in the main figures were performed on OFF responses; parallel analyses for ON responses are shown in fig. S3. Receptive field centers were defined as the center of mass of each ROI’s spatial profile. Receptive field offset was defined as the absolute distance between this center and the anatomical position of the ROI. Receptive field size was calculated as the full width at 25% of the peak amplitude after aligning profiles to their centers. Because the receptive field mapping stimulus varied positions only along the horizontal axis, we restricted our analysis of distal dendrites to those approximately aligned with this axis (ROIs oriented within ±45° of the stimulus axis). Response profiles and population data for vertically oriented distal dendrites (|dendrite angle| > 45°) are shown in fig. S3. Data for receptive field mapping were collected from 11 imaging regions across four mice.

To probe long-distance integration, we presented concentric contrast-inverting bright and dark rings on a gray background. Ring widths were fixed at 37.5 μm; inner diameters ranged from 0 to 750 μm in 150-μm steps (outer diameter = inner diameter + 150 μm). We measured the peak Δ*F*/*F* for each ring diameter and normalized responses to the maximum across diameters. The effective integration diameter was defined as the largest inner diameter that elicited >25% of the maximal response. Data were collected from four imaging regions in four mice (control) and four imaging regions in two mice (TTX).

### Orientation tuning analysis

To probe orientation tuning, we first identified the receptive field center of the image frame by presenting a 50-μm square at positions from −100 to +100 μm (25-μm increments) and determining the coordinates of maximal TH2 plexus responsiveness. We then presented stationary oriented bars (800-μm height, 50-μm width) centered at these coordinates. Bars were presented at multiple orientations for 3-s ON and 3-s OFF. To quantify orientation tuning, we computed the global Orientation Selectivity Index (OSI) and the preferred orientation (θ_pref_) using a vector-sum average of responses to oriented bars. For each ROI, we calculated the complex response vector *L*

$$L=\sum R\left(\theta \right)e^{2i\theta }$$

where *R*(θ) is the mean response amplitude to the angle θ. The factor of 2 accounts for the 180° periodicity of orientation space. The OSI was defined as the magnitude of this vector normalized by the total response

$$\text{OSI}=\frac{|L|}{\sum R\left(\theta \right)}$$

ROIs with OSI > 0.3 were classified as strongly tuned. The preferred orientation was derived from the angle of the resultant vector

$$\theta_{\text{pref}}=\frac{1}{2}\text{Arg}\left(L\right)$$

Local dendrite orientation (θ_dend_) was estimated from the confocal skeleton. Alignment between the functional preference and anatomy was quantified as the absolute difference |θ_pref_ – θ_dend_|.

To determine the random baseline for tuning, we generated a null distribution for each ROI by randomly permuting the assignment of recorded response traces to stimulus angle labels 1000 times. This preserved the response amplitudes but disrupted their relationship to the stimulus orientations. To determine the random expectation for anatomical alignment, we compared the observed preferred orientations with uniformly distributed random angles (0° to 180°; 1000 iterations). Confidence intervals (95%) were calculated using bootstrap resampling with 10,000 iterations. For orientation tuning, data were collected from five imaging regions in three mice.

### Patch-clamp electrophysiology

Retinas were isolated from dark-adapted (>1 hour) wild-type mice under infrared illumination and mounted on poly-l-lysine–coated coverslips (Corning). The tissue was continuously perfused (∼7 ml/min) with warm (∼32°C) bicarbonate-buffered Ames’ medium (Sigma-Aldrich; equilibrated with 95% O_2_/5% CO_2_). Recordings were performed on a Scientifica two-photon microscope equipped with a MultiClamp 700B amplifier (Molecular Devices).

W3/UHD ganglion cells were targeted for whole-cell patch-clamp recordings under infrared illumination. Correct targeting was confirmed by matching spike responses to previously established W3/UHD tuning profiles and morphologically by the inclusion of Alexa 488 (0.1 mM) in the patch pipette solution and subsequent two-photon imaging.

For whole-cell voltage-clamp and cell-attached recordings, the patch pipette (4 to 7 MΩ) solution contained 105 mM Cs-gluconate, 20 mM Na-Hepes, 10 mM EGTA, 10 mM TEA-Cl, 2 mM QX-314, 5 mM adenosine 5′-triphosphate–Na, and 0.1 mM guanosine 5′-triphosphate–Na (pH adjusted to 7.2 with CsOH). Patch pipettes were pulled to resistances of 4 to 7 MΩ. Series resistance (10 to 15 MΩ) was compensated by ∼75%. Cells were voltage clamped at 0 mV to record IPSCs. Signals were filtered at 3 kHz (8-pole Bessel low-pass), sampled at 10 kHz, and analyzed using pClamp 10 (Molecular Devices).

### Visual stimulation—Patch-clamp electrophysiology

Visual stimuli were written in MATLAB using Cogent graphics extensions (J. Romaya, University College London, London, UK) and delivered via an E4500 MKII PLUS II projector illuminated by a 385-nm ultraviolet LED (EKB Technologies). Stimuli were focused on the photoreceptors through the substage condenser and centered on the soma of the recorded cell. Projector output was attenuated with neutral density filters and calibrated using a spectrometer (BLACK-Comet, StellarNet) and a photometer (S450, UDT). The mean intensity of visual stimuli in patch-clamp recordings was 1500 R* and 314 S*.

### Surround suppression analysis

To measure the spatial extent of inhibition, we recorded IPSCs in voltage-clamp mode while presenting white annuli on a gray background (1-s ON and 1-s OFF). Annuli had a fixed width of 37.5 μm and inner diameters ranging from 0 to 1350 μm. For the smallest stimulus (inner diameter: 0 μm), the annulus effectively formed a 150-μm-diameter spot.

To test surround suppression of spiking, we recorded loose-patch spike responses to a 25-μm-diameter central spot presented alone or in combination with annuli. We used the same annular dimensions as above, with inner diameters varying from 0 to 1050 μm. In this protocol, the first two conditions corresponded to a 25-μm spot alone (0 μm-inner-diameter annulus) and a 150-μm spot (25–μm–inner-diameter annulus + 25-μm spot), respectively, while the remaining conditions consisted of the 25-μm central spot presented alongside annuli of increasing inner diameter. IPSC data using these stimuli were collected from seven W3/UHD ganglion cells, and spike data from loose-seal recordings were collected from 10 W3/UHD ganglion cells from four mice.

### Spatial integration of inhibition analysis

To test for linear integration within dendritic subunits, we presented white squares (100 × 100 μm) along four radial axes (45°, 135°, 225°, and 315°). We probed radial summation by presenting cumulative stimulus combinations at defined distances from the recorded soma. The base stimulus (stimulus 1) consisted of a single square centered at 400 μm. Subsequent conditions progressively added squares to this baseline: Stimulus 2r added a square at 259 μm (totaling 259 and 400 μm); stimulus 3r added a square at 541 μm (259, 400, and 541 μm); and stimulus 4r added a square at 683 μm (259, 400, 541, and 683 μm). To test orthogonal integration, we presented a configuration of three squares (stimulus 3o) arranged perpendicular to the radial axis, all positioned at a fixed distance of 400 μm from the soma.

Stimuli were presented for 1-s ON and 1-s OFF, with three repeats per condition interleaved in a pseudorandom order. To assess linearity, we also recorded responses to each square presented individually. We calculated the predicted linear response for the combined stimuli by summing the trial-averaged traces recorded for the constituent squares. Data were collected from seven W3/UHD ganglion cells across three mice. Because voltage clamp at the soma suppresses postsynaptic conductance interactions in W3/UHD dendrites, the spatial integration measurements reflect presynaptic interactions in the TH2 plexus.

### Motion density and coherence selectivity analysis

To probe object motion detection across a range of object densities, we created composite stimuli consisting of a central “probe” object and a surrounding context of incoherent motion (movie S1). The surround comprised incoherent moving objects (dark squares; 100 × 100 μm) traveling along linear trajectories at a constant speed of 500 μm/s within an annular region (inner boundary diameter: 300 μm; outer boundary diameter: 1595 μm). Surround objects reflected upon contacting the inner or outer boundaries, preserving a continuous field of incoherent surround motion while leaving the receptive field center void of background clutter. To test the resilience of W3/UHD responses to visual clutter, we systematically varied the density of these incoherent dark objects across trials, presenting 0, 7, 15, 23, 47, or 71 moving squares, corresponding to visual field occupancies ranging from 0.5 to 36%. Overlaid on this background, a single dark probe object (100 × 100 μm) traversed the receptive field center at 500 μm/s. The probe appeared every 3 s along randomized angles (0° to 360°) across a continuous 30-s trial.

To test responses to coherent global motion, we presented a dynamic wrapping grid of dark squares (100 × 100 μm) spanning the 1595-μm circular arena (movie S1). Grid spacing was scaled to match the equivalent densities tested under the incoherent condition. Stimuli were presented across six consecutive 5-s epochs. Each epoch consisted of a 3-s stationary phase followed by a 2-s motion phase, during which the entire grid translated coherently along a unified, randomized trajectory at 500 μm/s, driving simultaneous, correlated activation of the center and surround. As an additional coherent control condition (fig. S7), we presented a single large moving square (400 × 400 μm) occupying 8% of the visual field. All conditions were presented at a 60-Hz frame rate against a uniform gray background. Data quantifying maximum spike rates during incoherent motion were pooled from nine W3/UHD ganglion cells across three retinas, while data for coherent motion were collected from five W3/UHD ganglion cells across two retinas.

### Spike analysis

Spike times were binned at 50-ms intervals (20 Hz) to construct peristimulus time histograms (PSTHs). Peak firing rates were defined as the maximum bin value in the PSTH, averaged across three repeats per stimulus condition. For population comparisons of incoherent and coherent motion across stimulus densities, peak firing rates for each cell were normalized to its baseline response evoked by the central probe presented alone (0.5% visual field occupancy condition).

### IPSC analysis

Inhibitory currents were averaged across three repeats per condition. Peak amplitude was defined as the maximum outward current during the ON or OFF phase. For spatial integration experiments, we calculated the total inhibitory charge transfer as the area under the curve (AUC) of the IPSC trace. Linearity of summation was assessed by comparing the observed charge transfer in response to multisquare stimuli with the arithmetic sum of the charge transfers recorded for the constituent squares presented individually. For the comparison of radial versus orthogonal integration, responses were normalized to the charge evoked by the single proximal square (stimulus 1). To quantify the selectivity for surround geometry, we calculated a Radial Preference Index (RPI)

$$\text{RPI}=\frac{Q3_{r}-Q3_{o}}{Q3_{r}+Q3_{o}}$$

where *Q*3_r_ and *Q*3_o_ represent the inhibitory charge transfer evoked by the three-square radial and orthogonal configurations, respectively.

### Compartmental modeling

#### Morphology and discretization

We constructed a realistic compartmental model of the TH2 amacrine cell using the NEURON simulation environment accessed via its Python interface. The dendritic morphology was derived from a confocal reconstruction of a representative TH2 cell selected from our seven reconstructions and converted into standard SWC format. Because light microscopy limits the resolution of fine neurite caliber, dendritic diameters were assigned on the basis of our ultrastructural measurements from the 3DEM dataset. We sampled dendritic radius values as a function of distance from the soma in our 3DEM dataset and extrapolated this curve to assign realistic dendritic radius values to the reconstructed cell, resulting in a median radius of 0.19 μm. To ensure high spatial accuracy for calculating electrotonic signal propagation, the morphology was discretized into compartments such that no segment exceeded 1 μm in length.

Inhibitory currents were averaged across three repeats per condition. Peak amplitude was defined as the maximum outward current during the ON or OFF phase. For spatial integration experiments, we calculated the total inhibitory charge transfer as the time integral (AUC) of the IPSC trace. Linearity of summation was assessed by comparing the observed charge transfer in response to multisquare stimuli with the arithmetic sum of the charge transfers recorded for the constituent squares presented individually. For the comparison of radial versus orthogonal integration, responses were normalized to the charge evoked by the single proximal square (stimulus 1).

#### Synaptic distribution and mechanisms

We distributed excitatory synaptic inputs across the dendritic arbor to mimic the innervation pattern of OFF bipolar cells. On the basis of our CLEM quantification, synapses were placed at a uniform density of 0.064 syn/μm along the path length of all dendritic segments. Synaptic events were modeled using a biexponential conductance change (Exp2Syn) with rise and decay time constants of 1 ms (τ_1_ = τ_2_ = 1 ms) and a reversal potential of 0 mV, consistent with the fast kinetics of AMPA/kainate receptors.

#### Biophysical mechanisms

To simulate dendritic integration, we incorporated both passive and active membrane properties. All compartments were assigned a specific membrane capacitance (*C*_m_) of 1 μF/cm^2^, an axial resistivity of 60 Ω · cm, and a passive leak conductance *g*_leak_ with a reversal potential *E*_leak_ of −55 mV. Although TH2 cells are nonspiking interneurons, our pharmacological results indicated a role for voltage-gated sodium channels in signal propagation. We therefore modeled a sodium conductance *g*_Na_ using a Hodgkin-Huxley formalism adapted from previous models of amacrine cells (*43*, *44*). The membrane voltage *V* at position *x* and time *t* was evolved according to the cable equation

$$C_{m}\frac{\partial V}{\partial t}=\frac{d}{4R_{a}}\frac{\partial^{2}V}{\partial x^{2}}-g_{\text{leak}}\left(V-E_{\text{leak}}\right)-I_{\text{Na}}-I_{\text{syn}}$$

where *d* is the dendritic diameter and *R*_a_ is the axial resistance. The sodium current *I*_Na_ was defined as

$$I_{\text{Na}}={\bar{g}}_{\text{Na}}m^{3}h\left(V-E_{\text{Na}}\right)$$

where ${\bar{g}}_{\text{Na}}$ is the maximal sodium conductance density, *E*_Na_ is the sodium reversal potential (50 mV), and *m* and *h* are the voltage-dependent activation and inactivation gating variables, respectively.

To minimize unconstrained free parameters and isolate the specific interactions between branching geometry, passive cable properties, and sodium-mediated amplification, we assumed a uniform subcellular distribution of *g*_Na_ across the arbor and modeled synaptic events with fixed biexponential kinetics. While other active conductances (such as voltage-gated potassium or calcium channels) or nonuniform channel clustering may contribute to dendritic integration in vivo, restricting our model to mechanisms directly constrained by our empirical TTX validation datasets was sufficient to robustly replicate the observed proximal-distal receptive field dichotomy.

#### Parameter tuning and model constraints

To constrain the free parameters (*g*_leak_, *g*_Na_, and synaptic weight), we performed a systematic grid search visualized as an optimization manifold. For every combination of *g*_leak_ and *g*_Na_, we adjusted the synaptic weight to ensure the peak dendritic depolarization reached a physiologically realistic target [−20- to −30-mV peak amplitude (*22*)] in response to a standard visual stimulus. This procedure isolated the specific contributions of membrane properties to signal propagation, independent of synaptic strength. To map these abstract conductance densities to a functional metric, we calculated the effective electrotonic length constant for each parameter set. This was achieved by applying the tuned *g*_leak_ and *g*_Na_ values to a simplified “straight cable” model with a diameter fixed to the median radius of the TH2 arbor (0.19 μm). λ was quantified from the spatial decay of the voltage profile along this infinite cable. This approach allowed us to simulate circuit performance across a controlled continuum of integration regimes, from local integration (short λ) to global integration (long λ). The model reproduced the receptive field size and offset preferences observed in our data across a range of length constants. For the remainder of the study, we selected a representative λ of 460 μm to model the physiological TH2 cell. The soma was modeled as a continuous cable integrated within the proximal arbor (fig. S4). Control simulations confirming that the soma does not act as an artificial charge sink and that removing it does not alter the proximal-distal receptive field dichotomy are detailed in fig. S5.

#### Receptive field simulation and analysis

To map the receptive field of individual dendritic segments, we simulated responses to a grid of visual stimuli that tiled the arbor. The stimulus consisted of 50 × 50 μm patches presented at 50-μm intervals. For each stimulus position, we activated all synapses within the patch, weighted by a Gaussian-smoothed spatial profile (σ = 25 μm) to approximate the receptive-field response of presynaptic bipolar cells. We recorded the peak voltage response at every dendritic segment for each stimulus location.

Receptive fields were reconstructed by mapping the peak voltage recorded at a specific segment back to the coordinates of the stimulus grid. We quantified the receptive field properties for each segment by thresholding the spatial response profile at 25% of the maximum amplitude. The receptive field size was defined as the major axis length of the thresholded region, and the receptive field offset was calculated as the Euclidean distance between the recording site (dendritic segment) and the center of mass of the receptive field.

The codebase for these simulations is available at https://github.com/pratyush-washu/th2_rf_simulation. This repository contains reconstructed TH2 morphologies (SWC format), NEURON model definitions (including .mod files for active conductances), and Python scripts for receptive field mapping. It also includes the complete three-stage pipeline for generating synaptic distributions, simulating dendritic integration, and analyzing receptive field properties for a single cell under defined biophysical parameters.

#### Modeling the role of inhibition

To test how the sparse inhibition characteristic of TH2 cells shapes spatial integration, we added inhibitory synapses to the model (Fig. 5). Consistent with the OFF dominance of TH2 responses and our focus on OFF receptive fields, excitatory inputs represented OFF bipolar cell synapses only, distributed at the density measured in our 3DEM reconstructions (0.064 syn/μm). Inhibitory synapses were distributed at the total density of inhibitory inputs onto TH2 dendrites (0.08 syn/μm). Because presynaptic reconstructions of the inhibitory inputs were unavailable, inhibitory synapses were modeled as transient ON-OFF conductances, consistent with the bistratified mid-IPL inputs that target the TH2 plexus. During receptive-field–mapping simulations, excitatory synapses within the local stimulus patch and all inhibitory synapses across the arbor were activated simultaneously; this reflects the relative spatial scales of the local excitatory stimulus and the broadly distributed inhibition rather than an assumption of cotiming at the single-synapse level. Inhibitory synaptic weights were set to one-tenth of the excitatory weight (*w*_inh_ = 0.1·*w*_exc_), and *g*_leak_ was rescaled to preserve the physiological length constant (λ = 460 μm). OFF excitatory synapses were activated only within the local 50 × 50 μm stimulus patch, whereas all inhibitory synapses were activated simultaneously. Comparing the same arbor with and without this inhibitory input isolates the contribution of sparse inhibition to integration.

To assess how increased inhibition would affect dendritic integration, we subsequently replaced the TH2 synaptic densities with those of VG3 amacrine cells (excitatory: 0.035 synapses/μm; inhibitory: 0.169 syn/μm). Under these high-inhibition conditions, we observed that the receptive field size and offset responses resembled those of a cell with a much shorter length constant (λ = 200 μm), indicating that the low density of inhibitory inputs onto TH2 cells is consistent with their long-distance integration. To estimate the effective electrotonic length constant for these different conditions, we matched the resulting receptive field size and offset measurements to the reference curves established in Fig. 4B. We note that while the specific E/I weight ratio of 1:10 is an approximation, the qualitative collapse of the length constant under VG3-like inhibition densities provides a robust bound on the influence of lateral inhibition.

#### Sodium channel density and model fitting

While multiple combinations of leak (*g*_leak_) and sodium conductances (*g*_Na_) could reproduce the basic receptive field mapping (Fig. 3), precise quantification of active versus passive contributions required constraining the model to the more complex size-preference dataset (Fig. 6). To do this, we simulated the concentric ring protocol used in our two-photon imaging experiments by activating ON and OFF bipolar cell inputs within annuli of increasing diameter. We calculated size tuning curves for the proximal and distal compartments and systematically compared them with responses recorded under both control and TTX conditions. By identifying the parameter set that simultaneously reproduced the experimentally observed passive (TTX) and active (control) size preferences, we constrained the final conductance densities to *g*_leak_ = 1.2 mS/cm^2^ and *g*_Na_ = 1.9 mS/cm^2^. When applied to our simplified straight-cable model, these constrained values yielded effective electrotonic length constants of λ of 460 μm for the active (control) condition and λ of 110 μm for the passive (TTX) condition.

#### Role of branch pattern in compartmentalization

To investigate the contribution of dendritic morphology to the observed bipartite physiology, we constructed a synthetic wide-field amacrine cell with an inverted branching pattern (fig. S3). This “distal-branched” model matched the TH2 cell in total branch count and path length but featured a high density of distal branches rather than the proximal bias characteristic of TH2 arbors. We simulated receptive field mapping in this synthetic cell using the standard TH2 biophysical parameters (*g*_leak_ = 1.2 mS/cm^2^ and *g*_Na_ = 1.9 mS/cm^2^) derived from our model fitting.

In contrast to the TH2 cell, which exhibits two distinct functional compartments, the distal-branched morphology produced a tripartite functional organization (fig. S3). We observed small, local receptive fields in both the proximal and distal high-branching regions, while large, global receptive fields were restricted to the long, unbranched intermediate dendrites. These results indicate that high distal branching density creates local electrotonic compartments that shunt signal propagation. Thus, the specific “dense-proximal, sparse-distal” architecture of the TH2 cell is a critical structural determinant of its bipartite physiology, ensuring that distal neurites remain electrically compact to support global signal integration.

### Plexus modeling

#### Construction

We modeled the TH2 plexus in two dimensions over a 2 mm–by–2 mm field of view. To populate the model with TH2 cells, soma positions were identified from a confocal image of a TH-Cre retina (Fig. 2B; 634.8-μm field of view) to establish biologically realistic spacing. These soma coordinates were tiled to populate the full 2-mm field of view. We instantiated the “dense plexus” as a 3D NumPy array (2000 × 2000 × N), where each pixel corresponded to 1 μm^2^ of retinal space. To populate the dendritic plexus, one of the seven reconstructed morphologies was randomly selected for each soma location and assigned a random rotational angle. Dendritic coordinates from the corresponding SWC file were transformed by this rotation and placed relative to the soma center. Each TH2 arbor was stored in a separate layer of the 3D label matrix, with pixel values encoding both the source morphology ID and the path distance from the soma. An image of all TH2 cells whose dendrites enter a 150-μm-diameter region in the plexus is plotted in Fig. 7C; cells contributing proximal dendrites to this region are labeled in blue, and those contributing distal dendrites are labeled in orange. On average, 8.9 [8.4, 9.5] proximal and 86.8 [84.7, 88.8] distal TH2 cells contributed dendrites to a 150-μm-diameter region.

#### Overlap analysis and synapse sampling

We integrated a reconstructed W3/UHD RGCs into the dense plexus model. The W3/UHD morphology was rasterized into a 2D binary image, retaining only dendritic segments located within the stratification depth of the TH2 plexus. We computed the pixel-wise overlap between the W3/UHD mask and the TH2 dense plexus. Overlaps (appositions) were classified as “proximal” or “distal” based on the encoded distance in the TH2 pixel data (proximal < 76.1 μm; distal ≥ 76.1 μm). To model synaptic connectivity, a subset of appositions was stochastically labeled as synapses. The probability of a synapse forming at a given apposition was calibrated such that a single proximal TH2 cell formed approximately 10 inhibitory synapses with a downstream W3/UHD cell, matching our empirical observations. We placed the putative W3/UHD cell at multiple positions within the plexus to calculate connectivity statistics. On average, each W3/UHD cell received 38.5 [37.1, 40] proximal synapses (from 3.4 [3.1, 3.7] TH2 cells) and 209.2 [179.7, 238.6] distal synapses (from 38.5 [37.1, 40.0] TH2 cells). The W3/UHD cell, along with its proximal and distal inputs, is shown in Fig. 7D.

#### Receptive field reconstruction

To estimate the functional receptive field (RF) of the W3/UHD ganglion cell, we mapped the sampled synapse locations back to the spatial RFs of the presynaptic TH2 cells. For each inhibitory output synapse, we retrieved the spatial RF of the corresponding TH2 cell segment from the NEURON mapping. Specifically, we aligned the dendritic location of each synapse to the canonical morphology, selected the nearest RF subunit, and projected it back to the global coordinate frame. Figure 7 (E and F) shows example proximal and distal maps aligned to the coordinate frame of the downstream W3/UHD cell. This process was repeated for all proximal and distal synapses to generate a cumulative inhibition heatmap (Fig. 7G). For the simulations calculating the influence and number of synaptic contacts, we placed the W3/UHD cell in different regions of the plexus and averaged the values to ensure robustness; however, Fig. 7 depicts a single representative model instantiation to illustrate the spatial anisotropy inherent to the distal inhibitory surround.

Custom Python scripts used to generate the dense plexus model are available at https://github.com/pratyush-washu/plexus_model_git. This code includes routines for tiling the 2D soma distribution, instantiating the 3D voxelated forest from rotated morphologies, and calculating the probabilistic synaptic overlap between the TH2 plexus and postsynaptic W3/UHD ganglion cells.

### Statistical methods

Statistical analyses used both parametric and nonparametric approaches to estimate effect sizes. Unless otherwise stated, we report effect sizes with 95% confidence intervals [lower bound, upper bound]. For means, confidence intervals were calculated using the *t* distribution; for medians, bootstrap resampling with 10,000 iterations was used. For paired comparisons, we calculated confidence intervals for the ratio or difference of values. Confidence intervals for differences between independent groups were estimated using the Welch-Satterthwaite method, which accounts for unequal variance (scipy.stats.ttest_ind with equal_var = False). To determine whether observed connectivity between TH2 cells and RGCs exceeded predictions from chance overlap, we used a Monte Carlo simulation approach: Synapses were reassigned 10,000 times based on depth-overlap probability profiles derived from our 3DEM dataset and EyeWire reference data (*45*), with confidence intervals representing the central quantiles (0.5 to 99.5%) of ranked outcomes. To assess orientation tuning and anatomical alignment, null distributions were generated by drawing random dendritic angles from a uniform distribution (0° to 180°; 1000 iterations). No statistical methods were used to predetermine sample sizes. Data collection was not performed blind to experimental conditions, except where automated algorithms (e.g., shuffling) minimized bias. However, during manual segmentation of two-photon imaging data, proximal and distal ROIs were classified on the basis of morphology alone, blind to functional responses.
