# Supplementary material for: Population-level geometric filtering generates motion-coherence selectivity in the retina
Source: Sci Adv. 2026 Aug 19;12(34):eaeg7846. doi: 10.1126/sciadv.aeg7846 (PMC13488909; doi:10.1126/sciadv.aeg7846)
Supplement: Supplementary file 1 [file sciadv.aeg7846_sm.pdf]

Science Advances
AAAS

Supplementary Materials for

Population-level geometric filtering generates motion-coherence selectivity in the retina

Pratyush Ramakrishna *et al.*

Corresponding author: Josh L. Morgan, jlmorgan@wustl.edu; Daniel Kerschensteiner, kerschensteinerd@wustl.edu

Sci. Adv. **12**, eaeg7846 (2026)
DOI: 10.1126/sciadv.aeg7846

The PDF file includes:

Figs. S1 to S7
Legend for movie S1

Other Supplementary Material for this manuscript includes the following:

Movie S1

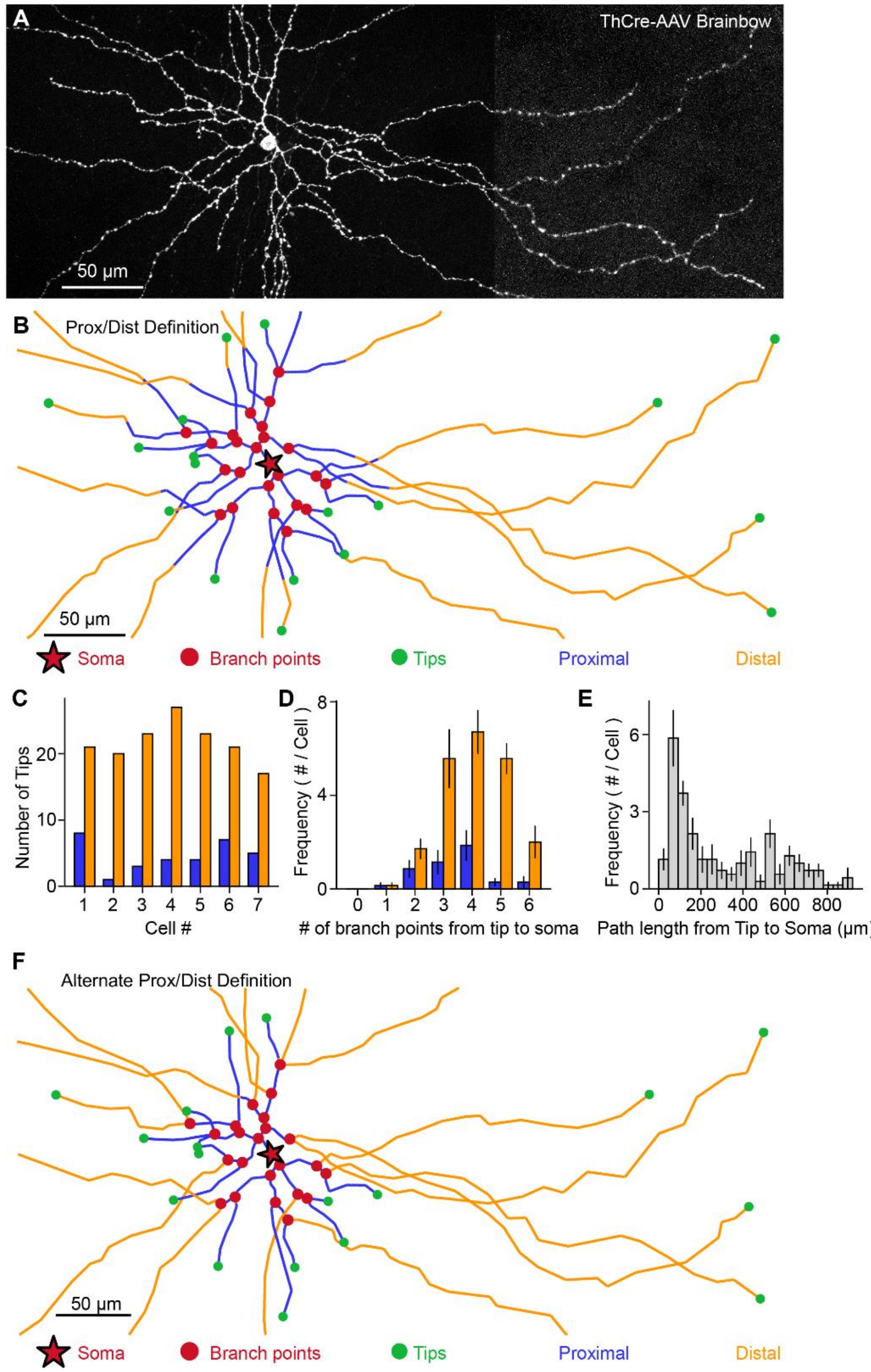

Fig. S1. Proximal dendrites branch successively to give rise to radiating distal dendrites. (**A**) Confocal projection of a sparsely labeled TH2 amacrine cell arbor illustrating branching density. (**B**) Morphological definitions used for proximal and distal segmentation of the reconstructed cell in (A). The soma (red star), branch points (red circles), and terminal tips (green circles) are identified from the confocal z-stack. Dendritic regions are classified as proximal or distal based on the distance to the closest branch point exceeding the 44.1 μm threshold established in Fig. 1. (**C**) Number of terminal tips located within proximal versus distal regions across reconstructed arbors (n = 7 cells). The majority of dendritic tips terminate in the distal region. (**D**)

Frequency distribution of the number of branch points encountered along the shortest path from each terminal tip to the soma. Together with (C), these data indicate that proximal dendrites undergo successive branching events to generate distal dendrites, rather than distal dendrites emerging directly from the soma. (**E**) Distribution of path lengths from terminal tips to the soma. While a subset of dendrites terminates close to the soma (<200 µm), a substantial fraction of tips per cell extends >400 µm. (**F**) Alternate morphological definition for proximal and distal segmentation, where the distal compartment is defined as extending from the final branch point along a path to a terminal tip. The distributions shown in (C-E) remain unchanged across both proximal/distal definitions.

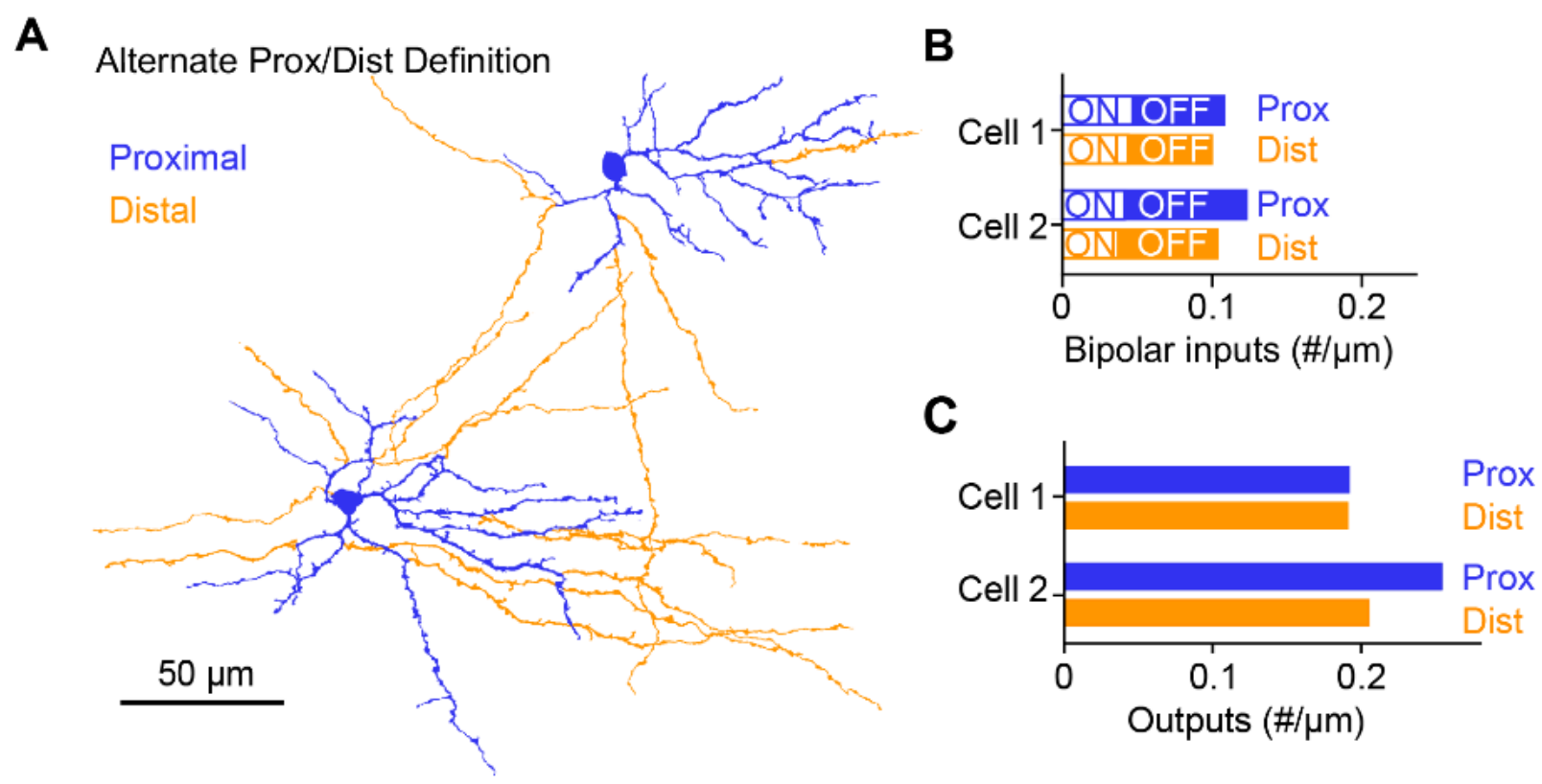

Fig. S2. Proximal and distal synaptic densities remain uniform under the alternate morphological definition.
(**A**) Ultrastructural reconstructions of the two TH2 amacrine cells from Fig. 2 re-segmented using the alternate definition established in Fig. S1, F. Dendritic regions extending from the final branch point along a path to a terminal tip are classified as distal (orange), while the remaining arbor is classified as proximal (blue). (**B** and **C**) Density of bipolar cell input synapses (B) and inhibitory output synapses (C) across proximal and distal compartments for both reconstructed cells. Consistent with the primary morphological definitions, both excitatory inputs and inhibitory outputs remain uniformly distributed across regions under this re-segmentation, confirming that synaptic uniformity is independent of the specific boundary used to segment the bipartite arbor.

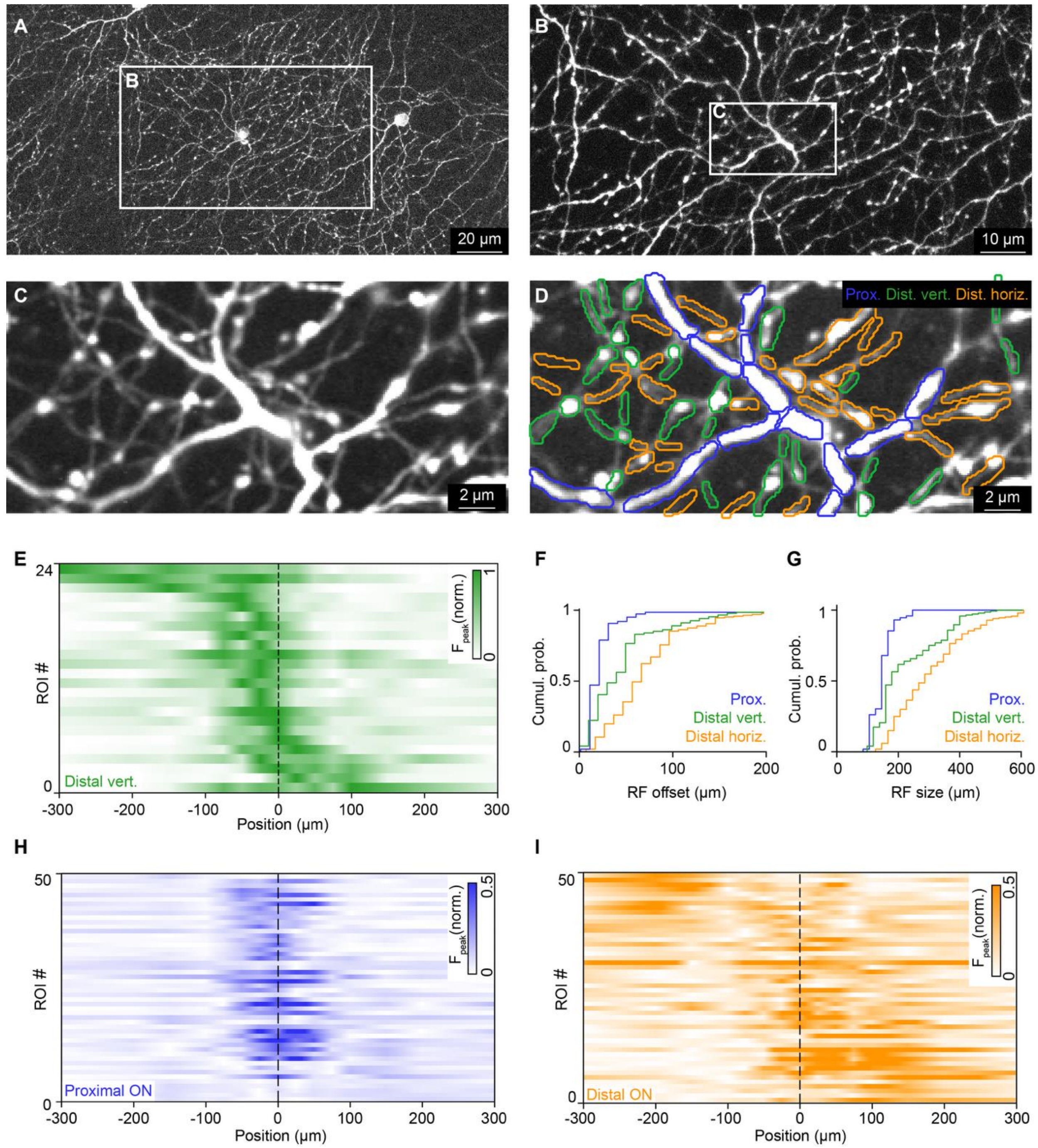

Fig. S3. Two-photon segmentation pipeline and functional mapping controls for proximal and distal dendrites.

(**A** and **B**) Representative two-photon z-stacks of the imaged TH2 plexus acquired at multiple zooms and spatial resolutions to establish morphological context. (**C** and **D**) Example segmentation of proximal and distal regions of interest (ROIs). Panel (C) shows the time-averaged fluorescence of the imaged region during visual stimulation. Typically, an individual imaging field captures a single soma and its highly branched proximal dendrites alongside multiple unbranched distal dendrites originating from neighboring cells. Using the multi-resolution z-stacks for verification, the soma and its tightly branched dendrites are classified as proximal (blue contours

in D), while unbranched processes traversing the field of view that adhere to the >44.1 μm inter-branch distance threshold are classified as distal. Horizontally and vertically oriented distal ROIs are indicated by orange and green contours in (D), respectively. (**E**) Peak-normalized OFF response amplitudes across horizontal bar positions for vertically oriented distal ROIs. Because the receptive field mapping stimulus varied strictly along the horizontal axis, the primary analyses in Fig. 3 were restricted to horizontally oriented distal dendrites (ROIs with dendritic angles between -45° and +45° relative to the horizontal axis). Panel (E) illustrates the spatial response profiles for the remaining subset of vertically oriented distal dendrites. (**F** and **G**) Cumulative probability distributions of OFF receptive field offsets (F) and sizes (G) comparing proximal dendrites (blue), horizontally aligned distal dendrites (orange), and vertically aligned distal dendrites (green). Vertically oriented distal dendrites exhibit receptive field offsets and sizes that are intermediate between proximal and horizontal distal dendrites, an expected outcome given that the one-dimensional stimulus does not capture the full spatial extent of receptive fields aligned along the vertical axis. (**H** and **I**) Peak-normalized ON response amplitudes across bar positions for reliable proximal (H) and distal (I) ROIs. Consistent with the strong OFF bias of the TH2 plexus, ON responses in both compartments are substantially weaker and less reliable than the corresponding OFF responses.

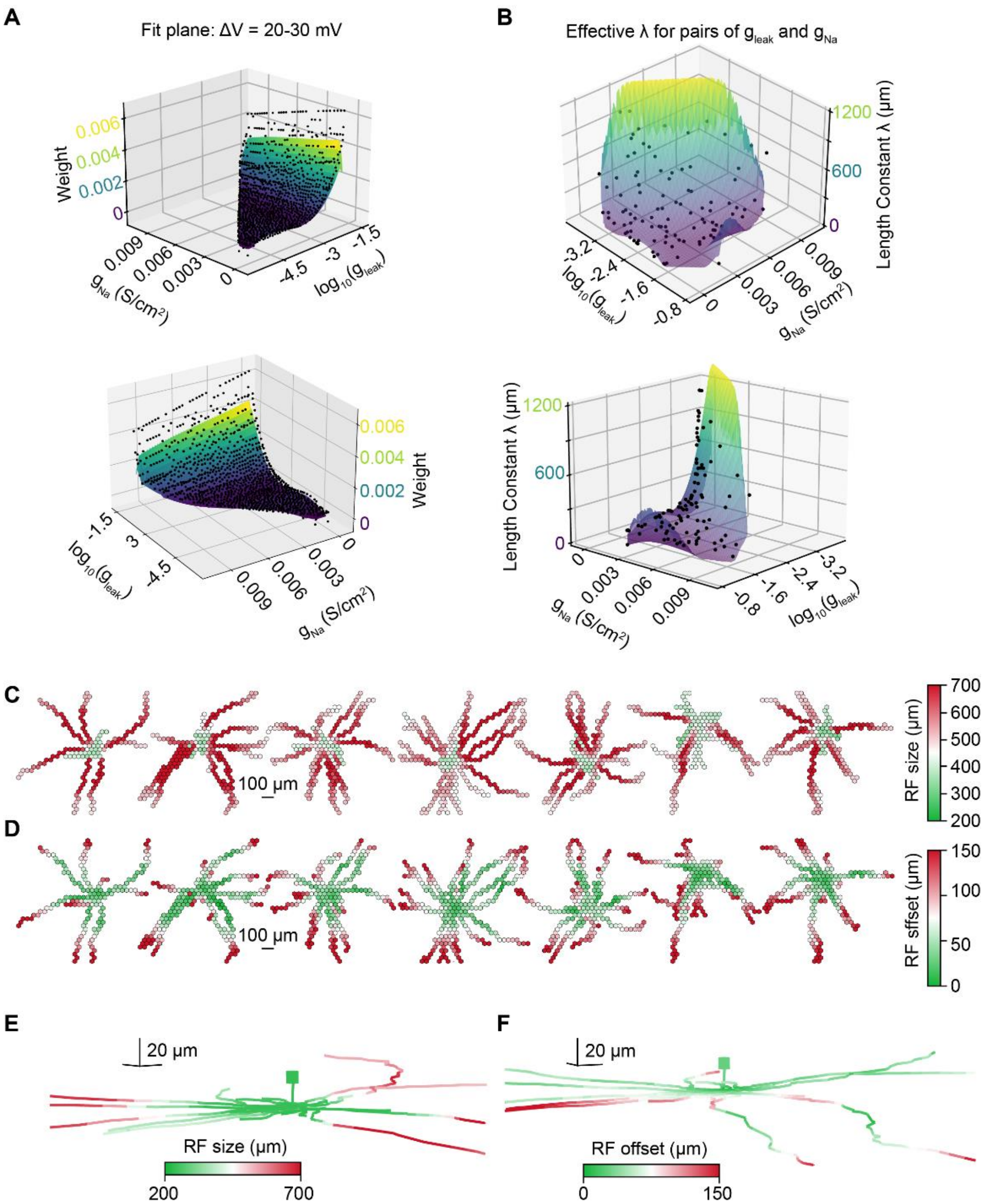

Fig. S4. Parameter tuning and model robustness.
(**A**) Optimization manifold visualizing the relationship between leak conductance g_{leak}, sodium channel density g_{Na}, and synaptic weight. Colored points indicate parameter sets that produced physiological depolarization amplitudes (20-30 mV), matching published patch-clamp data . (**B**) Determination of the effective electrotonic length constant (λ). Constrained parameter sets from (A) were applied to a simplified straight cable model to calculate λ values, which serve as the x-axis for the parameter sweep in Fig. 4B. (**C** and **D**) Simulated receptive field size (C) and offset (D) maps for all seven fully reconstructed TH2 cells using the optimized physiological parameters (λ of 460 μm; g_{leak} = 0.36 mS/cm^2, g_{Na} = 0.16 mS/cm^2). The characteristic bipartite physiology is conserved across all seven modeled cells. (**E** and **F**) Three-dimensional profile views of a TH2 cell illustrating receptive field size (E) and offset (F). The soma functions as a continuous, un-

isolated cable integrated directly within the proximal arbor, maintaining receptive field sizes and spatial offsets consistent with proximal dendrites.

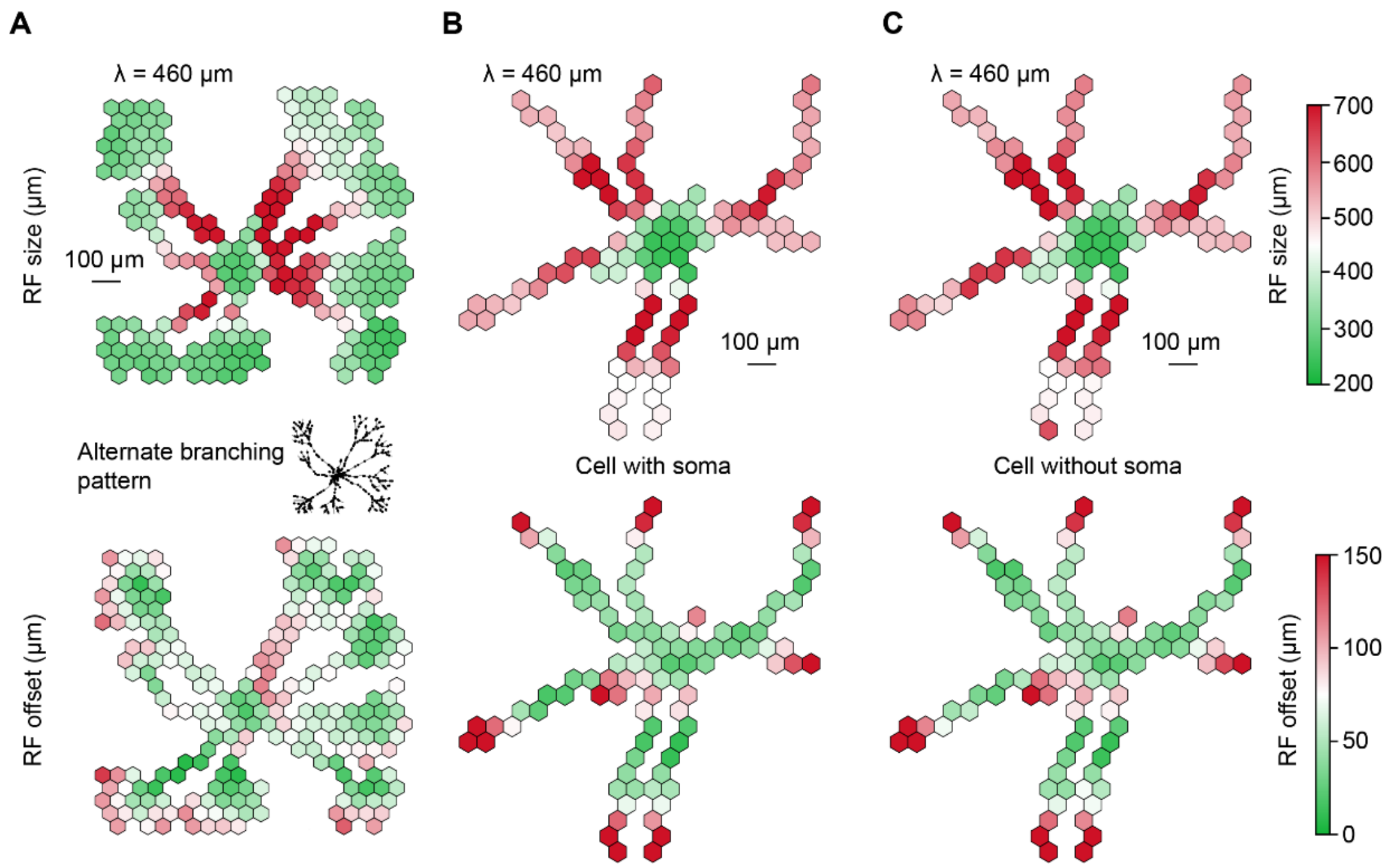

Fig. S5. Dendritic branching pattern determines functional compartmentalization.
(**A**) Receptive field size (top) and offset (bottom) maps simulated for a synthetic wide-field amacrine cell constructed with an inverted branching rule (sparse proximal branching, dense distal branching; inset) using standard TH2 biophysical parameters λ = 460 µm. In contrast to the bipartite physiology of native TH2 cells, dense distal branching establishes a tripartite functional organization characterized by small, locally centered receptive fields emerging within the highly branched distal tips. These results demonstrate that the specific dense-proximal, sparse-distal architecture of the TH2 arbor is required to maintain electrically compact distal neurites for global signal integration. (**B** and **C**) Simulated receptive field size (top) and offset (bottom) maps for a native TH2 morphology modeled with the soma included (B) versus the soma removed (C). The spatial distributions of receptive field sizes and offsets remain identical across both conditions, confirming that the soma does not act as an artificial charge sink for the proximal arbor and that the observed functional dichotomy is driven fundamentally by dendritic geometry.

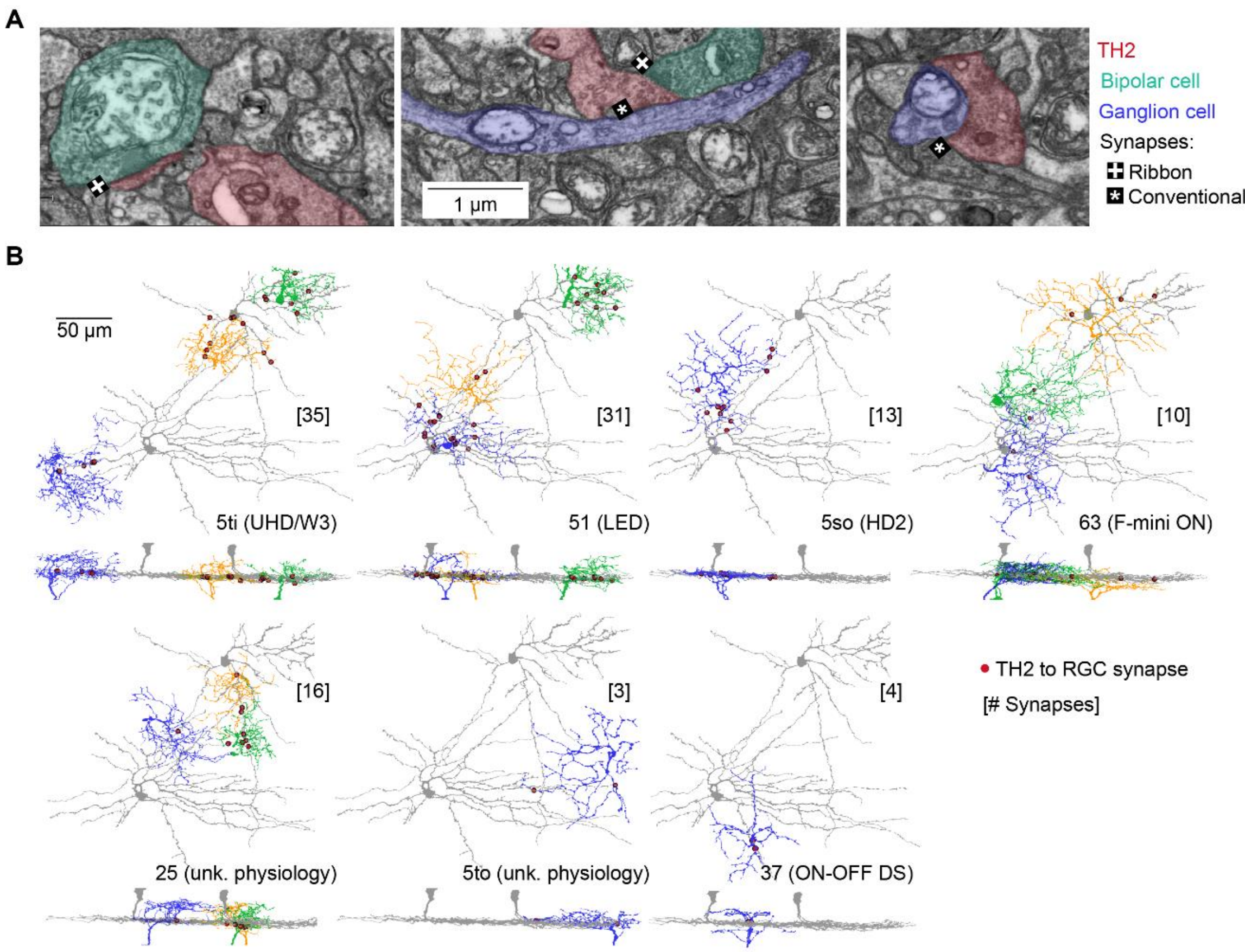

Fig. S6. Ultrastructural connectivity of the TH2 plexus.
(**A**) 3DEM segmentation of key synaptic motifs. Examples show an OFF bipolar cell ribbon synapse onto a TH2 dendrite (left), a dyad synapse where an OFF bipolar ribbon targets both a TH2 and a downstream ganglion cell to provide feedforward inhibition (center), and a standalone inhibitory synapse from a TH2 to a ganglion cell (right). (**B**) Inventory of all fully reconstructed ganglion cells postsynaptic to the TH2s in the volume, ordered by functional type. Colors indicate distinct cells, and numbers denote the total synapse count from the two annotated TH2 cells to each ganglion cell. RGC types were identified by comparing stratification profiles, soma size, and branching patterns with the EyeWire Museum database.

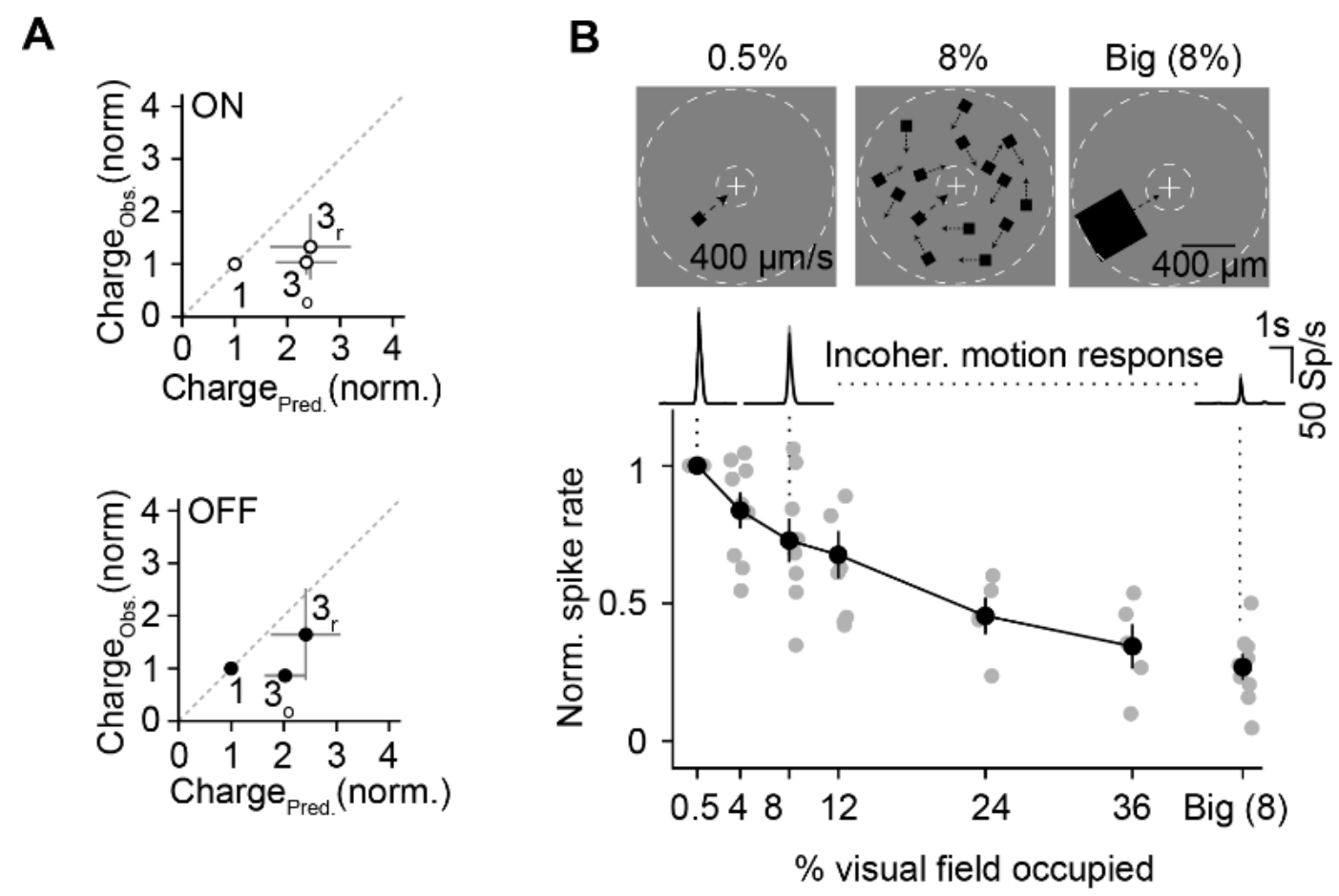

Fig. S7. Sublinear spatial integration of orthogonal inputs and population-level robustness to visual clutter.

(**A**) Observed versus predicted inhibitory charge comparing three squares arranged radially (3r) versus three squares arranged orthogonally (3o) for ON (top) and OFF (bottom) responses. Values are normalized to the single proximal square response (Stimulus 1), plotted on the same metric as Fig. 8, E. When composite responses are compared to their linear predictions (the arithmetic sum of individual constituent squares), radial configurations summate effectively, whereas orthogonal configurations evoke significantly weaker inhibition. (**B**) Population quantification mapping robustness to visual clutter. Top: Schematic of the probe plus clutter stimuli across increasing densities of incoherent surround motion (0.5-36% visual field occupancy) alongside a large-object motion control (400 µm square occupying 8% of the visual field). Middle: Example extracellular spike responses to the central probe object remain robust across clutter densities but are strongly suppressed by the large moving object. Bottom: Normalized peak firing rates across clutter densities and the large-object control condition (n = 9 cells from 3 mice). Spike rates are normalized to each cell's response to the central probe presented alone (0.5% occupancy condition), demonstrating that the TH2-mediated surround selectively filters large-object motion while preserving object detection across a wide range of object densities.

Movie S1. Visual stimuli used to test motion-coherence selectivity.
Representative incoherent and coherent motion stimuli used for the experiments in Fig. 8, G and H. In the incoherent condition, independently moving dark squares travel within an annular surround while a single dark probe traverses the receptive-field center. In the coherent condition, an array of identical dark squares translates collectively across the circular stimulus arena. Element size, speed, and visual-field occupancy were matched between conditions. Markers indicate the receptive-field center and stimulus boundaries.
